# Engrailed-1 potentiates mitochondrial transplant neuroprotection in spinal cord ischemia-reperfusion injury

**DOI:** 10.1038/s42003-026-10171-6

**Published:** 2026-05-07

**Authors:** Wei Wang, Rui Tang, Shilun Gao, Xiaotian Han, Teng Shi, Tianxiang Gu, Enyi Shi

**Affiliations:** 1 Department of Cardiac Surgery, First Affiliated Hospital, China Medical University, Shenyang, Liaoning China; 2 State Key Laboratory of Cardiovascular Disease, Center of Vascular Surgery, Fuwai Hospital, National Center for Cardiovascular Diseases, Chinese Academy of Medical Sciences and Peking Union Medical College, Beijing, China

**Keywords:** Neuronal physiology, Neurophysiology

## Abstract

Spinal cord ischemia–reperfusion injury (SCI/RI) triggers severe mitochondrial dysfunction and neuronal death. While mitochondrial transplantation (MT) is a promising strategy, its therapeutic potency remains limited. This study identifies the transcription factor Engrailed-1 (En-1) as a key regulator of mitochondrial homeostasis and a potential enhancer of MT. En-1 expression is significantly downregulated in SCI/RI models, whereas its restoration via hypoxic preconditioning or overexpression markedly improves neuronal survival. Mechanistically, En-1 stabilizes mitochondrial membrane potential, attenuates reactive oxygen species (ROS) production, and inhibits apoptosis by transcriptionally upregulating PDGFC. Mitochondria harvested from En-1-overexpressing cells (OE-En1-Mito) exhibit superior bioenergetic profiles and rapid neuronal uptake compared to unmodified mitochondria. In vitro, OE-En1-Mito increased ATP production and antioxidant activity; in vivo, transplantation preserved neuronal integrity and improved motor recovery in SCI/RI rats. Notably, silencing PDGFC in donor mitochondria abolished these neuroprotective benefits. Thus, En-1-modified MT provides superior neuroprotection for SCI/RI by leveraging the En-1/PDGFC axis.

**Figure:**
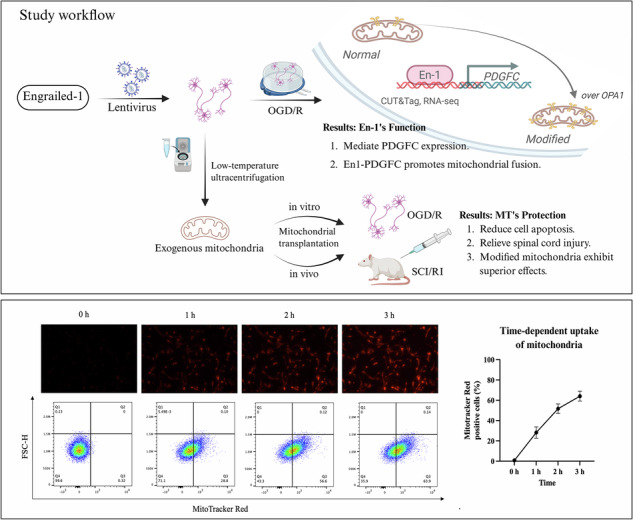

## Abstract (web summary)

Overexpression of Engrailed-1 in donor cells enhances mitochondrial transplantation’s efficacy by upregulating PDGFC-mediated mitochondrial fusion, offering a novel strategy to improve neuroprotection in spinal cord ischemia–reperfusion injury.

## Introduction

Spinal cord ischemia/reperfusion injury (SCI/RI) is a devastating complication of thoracoabdominal aortic surgeries, often resulting in paraplegia or death^1–3^. Current preventive measures, such as cerebrospinal fluid drainage, offer inconsistent efficacy, necessitating novel strategies to enhance spinal cord ischemic tolerance^4–7^. Mechanistically, SCI/RI triggers secondary injury cascades—including inflammation, oxidative stress, and apoptosis—centered on mitochondrial dysfunction^8–10^. Although mitochondria are pivotal regulators of energy metabolism and cell death^11,12^, pharmacological agents targeting mitochondrial oxidative damage or Ca^2+^ imbalance remain clinically unsatisfactory^13,14^. Therefore, directly preserving mitochondrial integrity represents a critical therapeutic critical for mitigating SCI/RI.

Mitochondrial transplantation (MT) represents an emerging therapeutic approach involving the transfer of healthy mitochondria into damaged tissues to restore bioenergetics^15,16^. Studies have demonstrated the efficacy of this method in restoring cell vitality following cardiac ischemia-reperfusion injury^17,18^ and improving neuronal survival under oxygen-glucose deprivation^19^. In this regard, recent strategies have focused on functionally enhancing mitochondria to augment these therapeutic effects. For instance, the pretreatment of mitochondria with Alda-1 before transplantation has been shown to enhance mitochondrial retention and inhibit apoptosis^20^. Alda-1 serves as an activator of aldehyde dehydrogenase 2 (ALDH2), which is an enzyme with a critical role in the maintenance of mitochondrial homeostasis in cardiomyocytes^21^. These observations suggest that the use of “gene-modified MT” constitutes a superior strategy for the treatment of SCI/RI.

Engrailed-1 (En-1) is a homeobox transcription factor known to play a major role in the nervous system^22–24^. Recent studies have demonstrated that neural stem cells upregulate En-1 expression under hypoxic conditions, which significantly attenuates apoptosis and promotes neuronal survival^25^. Additionally, En-1 exerts a protective effect against neuronal mitochondrial complex I dysfunction, thereby preserving essential cell survival pathways^26^. This cytoprotective capacity has been confirmed across diverse models, including malignancies and dopaminergic neurons. Specifically, En-1 inhibition was shown to trigger apoptosis while its overexpression enhanced resistance to mitochondrial inhibitors^27,28^. These findings indicate that En-1 increases cellular resilience against pathological insults. Given its high expression under hypoxia and its capacity to mitigate oxidative stress, En-1 is highly relevant to the pathophysiology of ischemia-reperfusion injury.

In this study, we investigated the regulatory role of En-1 in mitochondrial homeostasis using multi-omics sequencing. We aimed to elucidate the mechanisms by which En-1 suppresses oxidative stress-mediated apoptosis and facilitates functional remodeling in mitochondria. Furthermore, we developed a novel strategy for precisely restoring neuronal mitochondrial function involving the transplantation of “En-1-modified mitochondria”, thus providing new therapeutic insights for the prevention of ischemic SCI.

## Materials and Methods

### Animals and spinal cord ischemia

Adult male Sprague-Dawley rats (250–300 g) were utilized in this study. Animals were housed in a temperature-controlled facility under a 12-h light/dark cycle with ad libitum access to food and water. All procedures were approved by the Animal Management and Ethics Committee of China Medical University and conducted in accordance with the ARRIVE guidelines for reporting in vivo experiments^29^. Anesthesia was induced via isoflurane inhalation and maintained through an intraperitoneal injection of 2% sodium pentobarbital (2.5 ml/kg). At the experimental endpoint, rats were euthanized with an overdose of pentobarbital for spinal cord tissue harvesting.

The animal experiments were conducted in two distinct phases. In Phase I (target gene verification), rats were randomly assigned to a Control group or a SCI/RI group (*n* = 5 per group). In Phase II (mitochondrial transplantation), rats were randomly assigned to five groups (*n* = 10 per group): Control (Sham), SCI/RI, Mito-SCI/RI, Vector-Mito-SCI/RI, and OE-En1-Mito-SCI/RI. For transplantation, the 10 rats in each group were further subdivided into two cohorts (*n* = 5 per cohort) for histological analysis and behavioral assessments.

The spinal cord ischemia model was established as described in previously published studies (video available at: https://www.jtcvs.org/article/S0022-5223(18)32136-6/fulltext)^30–32^. The successful induction of the model was confirmed by the development of bilateral hindlimb paralysis shortly after reperfusion. Exclusion criteria included significant preoperative weight loss (>10%), signs of neurological deficits, or indications of systemic distress such as lethargy, abnormal posture, and piloerection. These criteria were applied to reduce variability and eliminate confounding factors that could independently influence neurological outcomes.

### Cell culture and OGD/R model

Highly differentiated PC12 cells, sourced from the Cell Bank of the Chinese Academy of Sciences, were maintained in DMEM (G4515, Servicebio, China) supplemented with 10% fetal bovine serum (F0193, Sigma-Aldrich, USA) and 1% penicillin-streptomycin (G4016, Servicebio, China). Cells were passaged or utilized for experiments upon reaching approximately 90% confluence.

To establish the Oxygen-Glucose Deprivation and Reoxygenation (OGD/R) model, the culture medium was replaced with glucose-free and serum-free medium. Cells were then incubated in a three-gas hypoxic incubator maintained at 37 °C with a continuous flow of hypoxic gas consisting of 95% N2 and 5% CO_2_. For hypoxic preconditioning, cells were treated for durations of 30, 60, 90, or 120 minutes. For the standard OGD/R model, cells underwent 6 hours of hypoxia followed by 24 hours of reoxygenation in complete medium under standard conditions of 37 °C and 5% CO_2_. All experimental groups were subsequently collected for downstream analysis.

### Cell viability assay

Cell viability was measured using cell counting kit-8 (CCK-8) reagent (G1613, Servicebio, China) according to the instructions. Cells were seeded in 96-well plates at a density of 1 × 10^3 cells/well. Measurements were performed for cells after hypoxic preconditioning and OGD/R injury, with three replicates per group. Then, 10 μL CCK-8 solution was added, and the cells were incubated at 37 °C for two hours. Finally, absorbance was measured at 450 nm using a microplate reader (Multiskan GO, Thermo Scientific, USA).

### Lentivirus and plasmid transfection

Rat Engrailed-1 lentiviral and control vectors were constructed by Syngentech (Beijing, China). PC12 cells were seeded at 1×10^6 per 10 mL and infected with En-1 or control vectors at a multiplicity of infection (MOI) of 20 for 24 hours. Successfully infected cells were selected using puromycin in complete medium. After 72 hours, fluorescence microscopy was used to monitor expression and cellular health. Transfection efficiency was validated via Western blot and RT-qPCR. For PDGFC knockdown, small interfering RNA (siRNA) was obtained from Thermo Scientific (USA), following the same transfection protocol as the lentiviral vectors.

### Integrated multi-omics and bioinformatics analysis

To investigate the regulatory network of En-1 and its impact on gene expression under OGD/R stress, we integrated CUT&Tag and RNA-seq technologies. Cell samples from the En-1 overexpression group were harvested, washed with PBS, and processed for chromatin extraction or total RNA isolation. All samples were promptly frozen at −80 °C prior to sequencing at Shanghai JiaYin Biotechnology.

For bioinformatic processing, raw reads were filtered using Trimmomatic, and uniquely mapped reads were utilized for subsequent analysis. For CUT&Tag data, peak calling was performed using MACS2, and HOMER was employed for motif discovery. For RNA-seq data, transcript counts were generated via HTSeq-count, and differentially expressed genes (DEGs) were identified using DESeq2 based on criteria of |log2FC | > 1 and *P* < 0.05.

Functional enrichment of these identified genes was performed using Gene Ontology (GO) and Kyoto Encyclopedia of Genes and Genomes (KEGG) databases. Significant categories were identified through Fisher’s exact test, and P-values were corrected using the false discovery rate (FDR) method. Detailed information regarding antibodies, specific software versions, and peak calling parameters is provided in the accompanying Reporting Summary.

### Dual-luciferase reporter assays

In order to verify the binding of the transcription factor EN1 (NM_001398705, CDS: 1230 bp) with the PDGFC promoter, the PDGFC promoter region was ligated into the pGL3-Basic vector. The HEK293T cells were prepared and transfected during the logarithmic growth phase. The following experimental groups were utilized:

- pGL3-Basic + pRL-TK Vector
- pGL3-Basic + EN1 + pRL-TK Vector
- pGL3-Basic-Promoter + pRL-TK Vector
- pGL3-Basic-Promoter + EN1 + pRL-TK Vector

Luciferase activity was detected using a kit (BL555A, Biosharp, China), with three parallel wells set for each group, and each well was tested three times.

### Transmission electron microscope

Cell pellets and isolated mitochondria were fixed in pre-cooled 2.5% glutaraldehyde and processed by Servicebio (Wuhan, China). Samples were dehydrated, embedded in epoxy resin, and ultrathin-sectioned to a thickness of approximately 70 nm. Mitochondrial morphology, including size, cristae integrity, and overall structural organization, was analyzed using a HITACHI HT7800 transmission electron microscope. Both intracellular and exogenous mitochondria were examined to evaluate morphological changes and structural integrity following injury.

### Detection of mitochondrial membrane potential

After cell seeding, OGD/R experiments were performed. The JC-1 probe was then used to assess the mitochondrial membrane potential (MMP) of live cells using the JC-1 MitoMP assay (G1515, Servicebio, China). The experimental results were then analyzed by flow cytometry (BD, FACSAria, USA) and fluorescence microscopy (DS-Ri2, Nikon, Japan).

### Mitochondrial isolation

Mitochondria were extracted from PC12 cells using the Cell Mitochondria Isolation Kit (C3601, Beyotime, China) according to the manufacturer’s protocol. Briefly, harvested cells were resuspended in isolation reagent and homogenized 30 times using a glass homogenizer on ice. The homogenate underwent differential centrifugation, including an initial spin at 1000 *g* for 10 minutes at 4 °C followed by a second spin of the supernatant at 3500 *g* for 10 minutes at 4 °C. The resulting mitochondrial pellet was resuspended in 100 μL of storage buffer and maintained on ice. To ensure functional integrity, all isolated mitochondria were utilized in subsequent experiments within 2–4 hours.

### Mitochondrial labeling and tracking

To differentiate mitochondrial origins during transplantation and in vivo injection, distinct fluorophores were utilized for labeling. Exogenous mitochondria were pre-incubated with MitoTracker Red (C1035, Beyotime, China) for 30 minutes at 37 °C prior to administration. Conversely, endogenous mitochondria were labeled with MitoTracker Green (C1048, Beyotime, China) under identical incubation conditions. Following the labeling process, fluorescent images were captured using a fluorescence microscope (DS-Ri2, Nikon, Japan). Mitochondrial fluorescence intensity and co-localization were subsequently quantified using ImageJ analysis software.

### Mitochondrial transplantation

In vitro, mitochondria were isolated using a standardized protocol, and uptake assays were conducted to determine the optimal co-culture duration for efficient internalization by recipient cells. To establish the appropriate transplantation dose, mitochondrial concentrations were quantified via protein assay. Based on previous studies^33–35^, the final dose was set at 100 μg/mL per well in a 6-well plate.

In vivo, preliminary intrathecal injections were performed to assess the uptake of exogenous mitochondria by spinal cord neurons and define the optimal time window for internalization. To minimize the risk of immune rejection, a concentration of 100 μg/μL was selected, with a total injection volume of 10 μL per animal. Mitochondria were administered intrathecally 24 hours prior to spinal cord ischemia-reperfusion surgery. For rats requiring hindlimb motor function evaluation, an additional injection was administered 3 days post-surgery at the same dose. The remaining animals commenced experimental procedures 24 hours after surgery.

### Annexin V-FITC/PI apoptosis detection

Logarithmically growing cultured cells were seeded in 6-well plates at a density of 2.0 × 10^5 cells per well. After attachment, the cells were subjected to OGD/R treatment. Cells treated with trypsin (without EDTA, G4002, Servicebio, China) were washed twice with PBS (G4202, Servicebio, China) and centrifuged. The cells were then incubated with 5 µL Annexin V-FITC staining solution and 5 µL PI staining solution (G1511, Servicebio, China) for 10 minutes in the dark. Data were analyzed by flow cytometry (BD, FACSAria, USA).

### Measurement of reactive oxygen species (ROS)

ROS levels were detected using DCFH-DA (G1706, Servicebio, China) according to the manufacturer’s instructions. The cells were seeded at appropriate density in 6-well plates and treated with OGD/R. Subsequently, the cells were exposed to DCFH-DA in the dark for 30 minutes. After the cells were collected, the results were measured by flow cytometry (BD, FACSAria, USA) and fluorescence microscopy (DS-Ri2, Nikon, Japan).

### Measurement of ATP, SOD, and MDA

After OGD/R treatment, cells were harvested for the following measurements. Adenosine triphosphate (ATP), superoxide dismutase (SOD), and malondialdehyde (MDA) concentrations were determined using the respective detection methods provided by the manufacturers. The results were analyzed to evaluate cellular energy metabolism, oxidative stress, and antioxidant capacity. The ATP detection kit (G4309) was purchased from Servicebio Biotechnology Co., Ltd. (China), while the SOD (WLA110) and MDA kits (WLA048) were obtained from Wanlei Biotechnology Co., Ltd (China).

### Activity assay of isolated mitochondrial respiratory complexes

The enzymatic activities of mitochondrial respiratory chain Complex I and Complex IV were measured in isolated mitochondria using their respective commercial assay kits (Complex I kit, P0423S, Beyotime, China; Complex IV kit, P0421S, Beyotime, China), following the manufacturers’ protocols. Specific activities were calculated and normalized to the total protein concentration of the mitochondrial preparations.

### Immunofluorescence (IF) analysis

PC12 cells were cultivated in 6-well plates at a density of 1.0 × 10^5 cells per well. The cells were fixed with paraformaldehyde and treated with Triton X-100 and goat serum for 30 minutes. The cells were then incubated with an anti-OPA1 antibody (1:1000, ab157457, Abcam, UK) at 4 °C. Subsequent to this, the samples were stained with a secondary antibody (FITC, WLA032, Wanleibio, China) and DAPI (P0131, Beyotime, China). Images were then captured using a fluorescence microscope (DS-Ri2, Nikon, Japan).

The spinal cord was fixed, paraffin-embedded, and incubated in serum solution. The tissue was then incubated with anti-Engrailed-1 (1:100, sc-398534 X, Santa Cruz, USA), anti-MAP2 (GB11128-2, Servicebio, China), and anti-GFAP (GB11096, Servicebio, China) antibodies. Subsequent to staining with secondary antibodies (iF647, G1232; iF488, G1231; iF546, G1251, Servicebio, China) and DAPI (G1012, Servicebio, China), the stained sections were imaged using a slide scanner (Pannoramic MIDI, 3DHISTECH, Magyar).

### Immunohistochemistry (IHC) analysis

The injured spinal cord tissue was meticulously collected, fixed, and embedded in paraffin. The tissue was then sectioned into 5 µm slices for staining. These sections were treated to restore antigenicity and suppress endogenous peroxidase activity. The cells were sealed in a serum solution and incubated with an Engrailed-1 antibody (1:100, sc-398534 X, Santa Cruz, USA). Stained sections were imaged using a slide scanner (Pannoramic MIDI, 3DHISTECH, Magyar). Selected images were analyzed using ImageJ.

### TUNEL apoptosis detection

Spinal cord tissue was harvested and subjected to TUNEL staining following 24 hours of spinal cord ischemia in rats. The tissue was processed according to the manufacturer’s instructions (G1502, Servicebio, China) following fixation and permeabilization. The tissue was then examined using a slide scanner (Pannoramic MIDI, 3DHISTECH, Magyar).

### Nissl body staining

In a manner analogous to the protocol employed for TUNEL staining, following fixation and permeabilization, the tissue underwent processing in accordance with the manufacturer’s guidelines (G1036, Servicebio, China). Subsequently, it was subjected to examination through the utilization of a slide scanner (Pannoramic MIDI, 3DHISTECH, Magyar).

### Western blotting analysis

Cells and isolated mitochondria were lysed, and protein concentrations were determined using a BCA assay (WLA001, Wanleibio, China). Equal amounts of protein were resolved by 10% SDS-PAGE (PG112, YAMAY BIOTECH, China) and transferred onto PVDF membranes (IPVH00010, Millipore®, USA). Membranes were blocked with QuickBlock™ (AR0041, BOSTER, China) for 20 min at room temperature, followed by overnight incubation with primary antibodies at 4 °C. After incubation with HRP-conjugated secondary antibodies (1:5000, WLA023, Wanleibio, China) for 2 hours, protein bands were visualized using ECL substrate (G2020, Servicebio, China) and captured with a chemiluminescence imaging system (ChemiDoc MP, Bio-Rad, USA). Detailed information regarding the primary antibodies used in this study is provided in the accompanying Reporting Summary.

### Quantitative real-time PCR (RT-qPCR)

Total RNA was extracted using the TRIzol method, followed by deoxyribonucleic acid (DNA) synthesis (RR047A, Takara, Japan) and quantitative polymerase chain reaction (qPCR) analysis (RR420A, Takara, Japan). Gene expression was determined using the 2-ΔΔCt method, which allows for comparison between samples. The primer sequences for qPCR are as follows:

*Engrailed-1* Forward: 5’- CTGGGTCTACTGCACACGCTACT -3’

*Engrailed-1* Reverse: 5’- CTCCGTGATGTAGCGGTTTG -3’

*PDGFC* Forward: 5’- GACAAGGAGCAGAACGGAGTG -3’

*PDGFC* Reverse: 5’- ATCTCCAGACCAGCACCGTATT -3’

*β-actin* Forward: 5’- TGCTATGTTGCCCTAGACTTCG -3’

*β-actin* Reverse: 5’- GTTGGCATAGAGGTCTTTACGG -3’

### Statistics and Reproducibility

All experimental data in this study were statistically analyzed using GraphPad Prism 8.0 software. Measurement data are expressed as mean ± standard deviation (SD), and data comparisons were performed using one-way ANOVA, with post-hoc analysis conducted using the Bonferroni correction method. A P value of less than 0.05 was considered statistically significant (* *P* < 0.05, ** *P* < 0.01, *** *P* < 0.001, **** *P* < 0.0001). For nonparametric data such as motor deficit index (MDI) scores and neuronal survival counts, the Mann-Whitney U test was used. Bonferroni correction was applied to adjust for multiple comparisons, with statistical significance set at P < 0.01.

### Reporting summary

Further information on research design is available in the Nature Portfolio Reporting Summary linked to this article.

### Ethics approval and consent to participate

All research protocols have been reviewed and approved by the Animal Ethics Committee of the Medical University. The animal care and use during the experiment were carried out in accordance with the guidelines of the Provincial Experimental Animal Management Committee.

## Results

### En-1 was downregulated in the rat SCI/RI model and conferred protective effects on mitochondrial function

#### SCI/RI leads to a decrease in En-1 expression

**Fig. 1:**
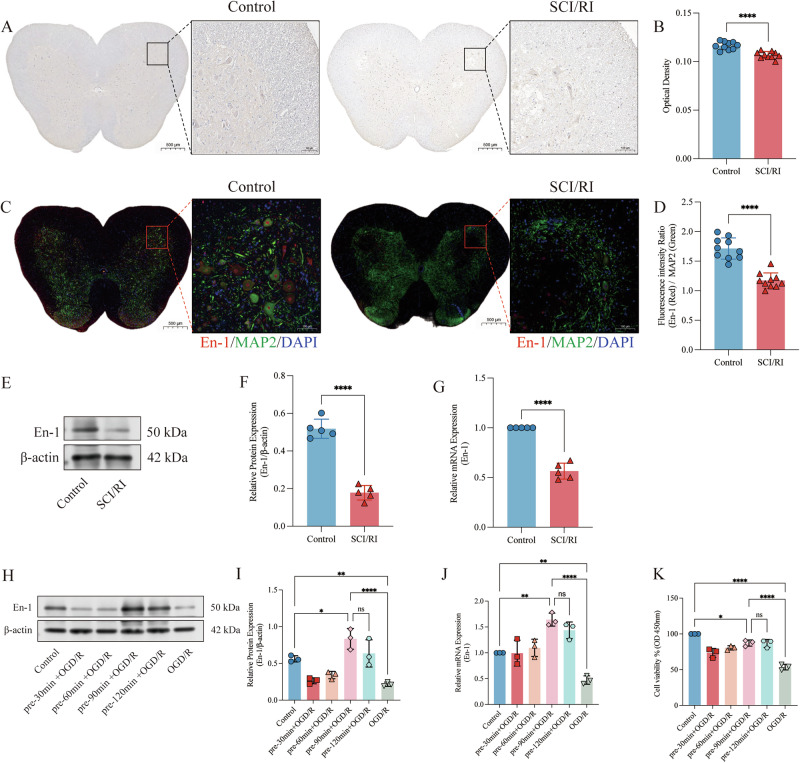
En-1 Expression Dynamics After SCI/RI and Hypoxic Preconditioning. **A**–**G** En-1 downregulation post-SCI/RI: (**A**–**D**) IHC, IF, and statistical analysis were used to quantify En-1 expression and observe its localization and distribution in tissue (*n* = 5). The green MAP2 is a neuron marker. Scale bars: 500 μm (whole spinal cord) and 100 μm (magnified views). **E**–**G** Western blot and RT-qPCR were performed to validate En-1 expression (*n* = 5). **H**–**I** Hypoxic preconditioning induces En-1: **H**–**J** Western blot and RT-qPCR showing time-dependent En-1 upregulation (30-120 min hypoxia. **K** CCK-8 assay showed changes in cell viability with increasing hypoxic preconditioning time (*n* = 3). All data are presented as mean ± SD for both graphs. Error bars indicate SD, and statistical analysis was performed using one-way ANOVA (* *P* < 0.05, ** *P* < 0.01, **** *P* < 0.0001).

To investigate En-1 expression in SCI/RI, we established a rat model and assessed En-1 levels via immunohistochemistry and immunofluorescence (Fig. 1A–D). Quantitative analysis of motor neuron-rich regions in the spinal cord anterior horn revealed a significant downregulation of En-1 post-injury (*P* < 0.0001). This reduction was further confirmed at both protein and mRNA levels using Western blot and RT-qPCR (Fig. 1E–G). These findings indicate that SCI/RI triggers a marked loss of En-1, suggesting its downregulation may contribute to the pathophysiology of spinal cord injury and providing a foundation for exploring its neuroprotective potential.

#### Hypoxic preconditioning enhanced cell viability by upregulating En-1

Studies have shown that hypoxic preconditioning typically induces a protective effect^36,37^. To explore the biological role of En-1, we subjected PC12 cells to various HPC durations (30, 60, 90, and 120 min). Western blot and RT-qPCR analyses revealed that En-1 expression increased in a time-dependent manner, peaking at 90 min (Fig. 1H–J, *P* < 0.01). Parallel CCK-8 assays showed that cell viability followed a similar trend, also reaching its maximum at the 90-min mark (Fig. 1K, *P* < 0.0001). Notably, the high consistency between En-1 levels and cell survival suggests that En-1 upregulation is a key mediator of hypoxic-preconditioning-induced protection. Furthermore, the significant En-1 downregulation observed in the OGD/R-only group (*P* < 0.01) reinforces its potential cytoprotective role during recovery from acute ischemic injury.

#### The overexpression of En-1 exerted cytoprotective effects and enhanced mitochondrial function

**Fig. 2:**
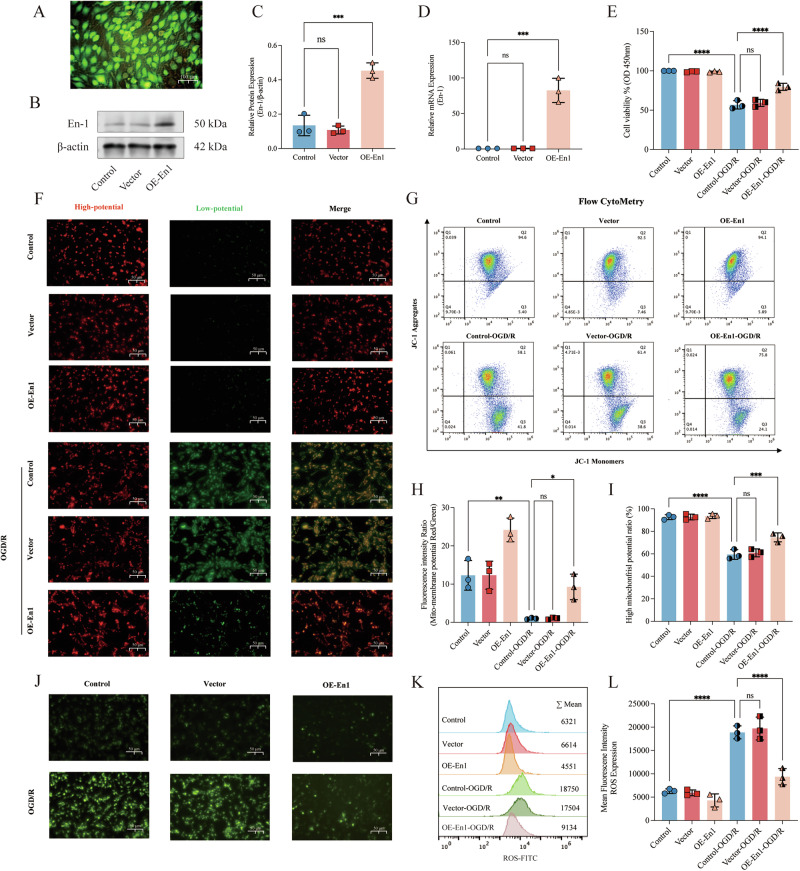
Constructing an En-1 overexpression stable cell line to demonstrate the protective effect of En-1 on mitochondrial function after OGD/R injury. **A** Fluorescence observation of transfection efficiency (MOI: 20). Scale bars: 100 μm. **B**–**D** Western blot and RT-qPCR confirmed the effective overexpression of En-1. **E** CCK-8 assay preliminarily demonstrated the protective effect of En-1 overexpression on cell injury. **F**–**I** Fluorescence images of JC-1 staining and flow cytometry analysis showing that OE-En1 cells maintain a higher mitochondrial membrane potential after OGD/R injury. Scale bars: 50 μm. **J**–**L** Fluorescence images of ROS staining and flow cytometry analysis indicating that the ROS level in the OE-En1-OGD/R group is significantly lower than in the Control-OGD/R group after injury. Scale bars: 50 μm. All data are presented as mean ± SD (*n* = 3) from three independent experiments. Error bars represent SD, and significance was assessed by one-way ANOVA (* *P* < 0.05, ** *P* < 0.01, *** *P* < 0.001, **** *P* < 0.0001).

To elucidate the biological function of En-1, we established a PC12 cell line stably overexpressing En-1 (OE-En1) via lentiviral infection, confirmed by fluorescence microscopy and validated by Western blot and RT-qPCR (Fig. 2A–D, *P* < 0.001). CCK-8 assays initially demonstrated that En-1 overexpression significantly enhanced cell viability against OGD/R-induced oxidative stress (Fig. 2E, *P* < 0.0001).

Given that mitochondria are central to energy metabolism and apoptosis, we evaluated whether En-1 confers neuroprotection by maintaining mitochondrial integrity. JC-1 staining and flow cytometry revealed that OE-En1 cells maintained significantly higher mitochondrial membrane potential following OGD/R injury compared to controls (Fig. 2F–I). Correspondingly, En-1 overexpression markedly suppressed ROS accumulation, as evidenced by both imaging and flow cytometric analysis (Fig. 2J–L, *P* < 0.0001), indicating robust protection of mitochondrial function.

**Fig. 3:**
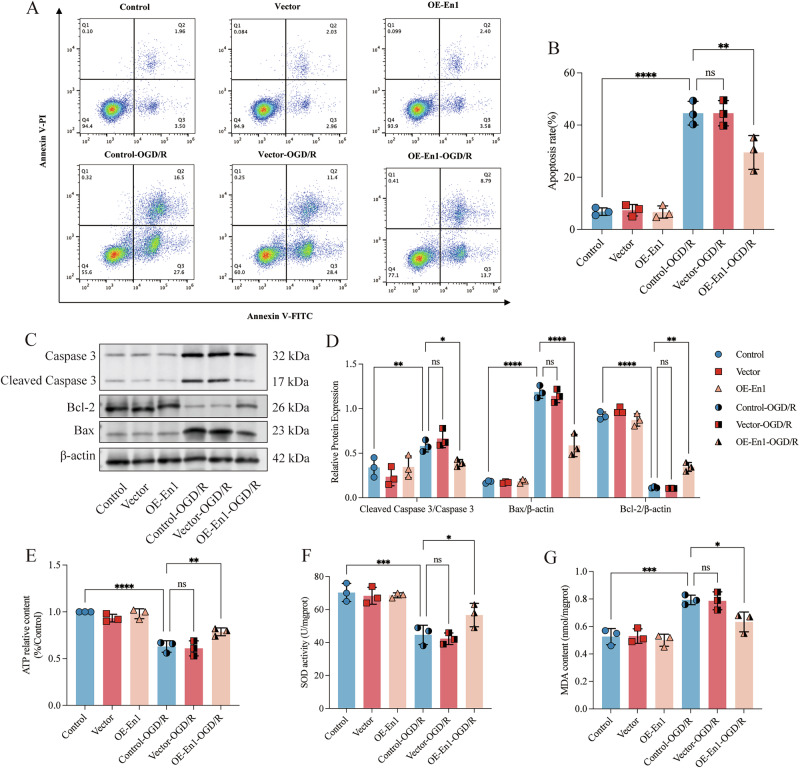
En-1 protects cells from apoptosis. **A**, **B** Annexin V flow cytometry analysis to detect cell apoptosis. **C**, **D** Western blot analysis to verify changes in apoptosis-related proteins. **E** ATP content measurement. **F** SOD activity assay. **G** MDA content measurement. All data are presented as mean ± SD (*n* = 3) from three independent experiments. Error bars represent SD, and significance was assessed by one-way ANOVA (* *P* < 0.05, ** *P* < 0.01, *** *P* < 0.001, **** *P* < 0.0001).

We further examined the impact of En-1 on programmed cell death. Annexin V-FITC/PI dual staining showed a significant reduction in apoptosis rates in the OE-En1 group post-OGD/R (Fig. 3A, B, *P* < 0.01). This was molecularly supported by the upregulation of the anti-apoptotic protein Bcl-2 and the concomitant downregulation of Bax and cleaved caspase-3 (Fig. 3C, D). Additionally, OE-En1 cells exhibited superior bioenergetic recovery and antioxidant capacity, characterized by increased ATP content and SOD activity, alongside reduced MDA levels (Fig. 3E–G). Collectively, these data substantiate that En-1 mitigates oxidative damage and inhibits mitochondria-dependent apoptosis by stabilizing mitochondrial homeostasis.

### En-1 Enhanced mitochondrial fusion via the modulation of PDGFC

#### Integrated CUT&Tag and RNA-seq analysis of the biological functions of En-1

**Fig. 4:**
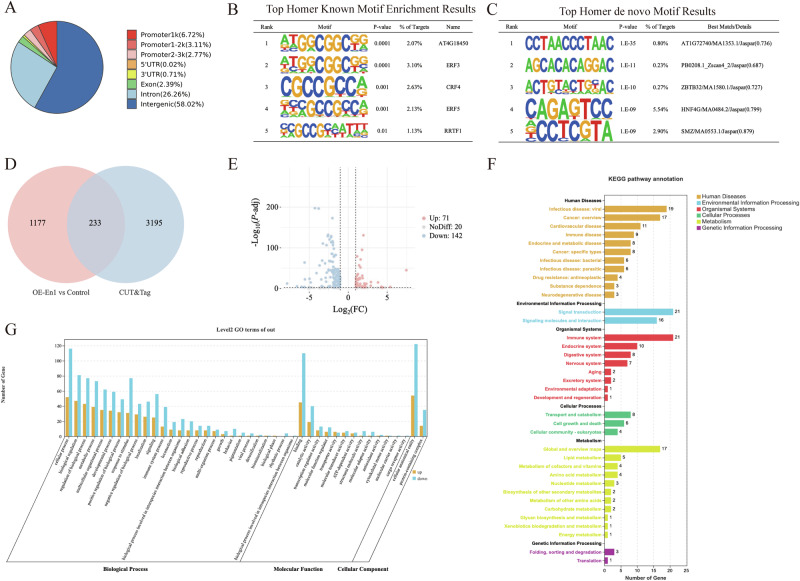
CUT&Tag (*n* = 1) and RNA-seq (*n* = 3) bioinformatics analysis to elucidate the biological function of En-1. **A** Peak annotation analysis. **B** Peak sequence Known Motif analysis. **C** Peak sequence de novo Motif analysis. **D** Venn diagram of integrated analysis. **E** Volcano plot. **F** GO analysis. **G** KEGG analysis.

To decipher the transcriptional landscape and molecular mechanisms governed by En-1, we performed integrated CUT&Tag and RNA-seq analyses. CUT&Tag profiling in En-1-overexpressing PC12 cells revealed that En-1 binding sites are predominantly distributed in intergenic regions (58.02%) and introns (26.26%), with 6.72% localized within 1-kb promoter regions (Fig. 4A). Motif enrichment analysis identified ERF3, CRF4, and ERF5 among the top enriched known motifs (Fig. 4B), suggesting potential co-regulatory interactions between En-1 and these factors. De novo motif analysis further highlighted the “CAGAGTCC” motif as a high-probability binding sequence (Fig. 4C), hinting at novel, non-canonical regulatory mechanisms. RNA-seq analysis was performed on normal cells and cells stably overexpressing En-1 (*n* = 3). Differential expression analysis (Log2FoldChange > 1, *P* < 0.05) revealed a distinct transcriptional shift following En-1 overexpression. By intersecting the CUT&Tag peaks with differentially expressed genes (DEGs), we identified 233 direct regulatory targets of En-1 (Fig. 4D, E).

Gene Ontology (GO) enrichment analysis of these targets (Fig. 4F) highlighted their involvement in critical biological processes, including signal transduction, immune modulation, and metabolic activities. Molecular function analysis showed significant enrichment in DNA-binding transcription factor activity and catalytic regulation, while cellular component analysis confirmed the localization of these targets to the nucleus, cell membrane, and mitochondria, reinforcing En-1’s role in mitochondrial homeostasis.

Furthermore, KEGG pathway analysis (Fig. 4G) revealed that En-1 targets are integrated into key signaling networks, including cardiovascular disease pathways, signal transduction, and global metabolic overview (lipid and nucleotide metabolism). Notably, the enrichment in environmental information processing and immune system pathways suggests that En-1 acts as a versatile regulator that maintains cellular homeostasis and bolsters adaptation to ischemic stress. These findings provide a comprehensive roadmap of the En-1 regulatory network and establish a mechanistic basis for its neuroprotective effects.

#### *PDGFC* was identified as a potential En-1 target gene in the regulation of mitochondrial fusion

**Fig. 5:**
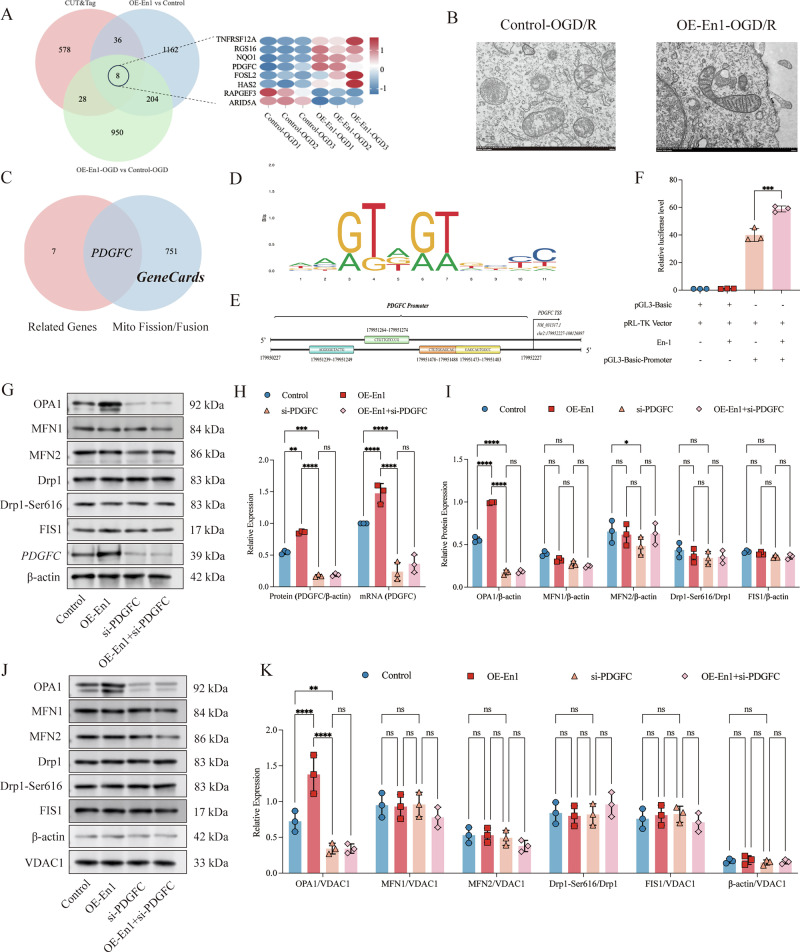
En-1 directly binds to and regulates PDGFC to mediate changes in mitochondrial fission and fusion. **A** Venn diagram to screen genes. **B** Transmission electron microscopy observation of mitochondrial fusion. Scale bars: 500 nm. **C** PDGFC identified as a key downstream gene of En-1. **D** Transcription factor En-1 Motif sequence diagram. **E** Schematic diagram of transcription factor En-1 binding to PDGFC. **F** Dual-luciferase assay confirming the positive regulation of PDGFC by transcription factor En-1. **G**–**I** Assessment of changes in the expression of key molecule and mitochondrial fission/fusion proteins in total cell lysates. **J**, **K** Confirmation of altered mitochondrial-localized fission and fusion protein expression in isolated mitochondria. All data are presented as mean ± SD (*n* = 3) from three independent experiments. Error bars represent SD, and significance was assessed by one-way ANOVA (* *P* < 0.05, ** *P* < 0.01, *** *P* < 0.001, **** *P* < 0.0001).

To pinpoint the key effectors of En-1, we integrated our multi-omics datasets with functional screening. Intersecting the differentially expressed genes from OGD/R-exposed OE-En1 cells with CUT&Tag-identified direct binding targets yielded eight candidate genes: *TNFRSF12A*, *RGS16*, *NQO1*, *PDGFC*, *FOSL2*, *HAS2*, *RAPGEF3*, and *ARID5A* (Fig. 5A).

Concurrently, transmission electron microscopy (TEM) revealed that OGD/R injury triggered severe mitochondrial fragmentation, characterized by membrane disintegration and cristae dissolution. Remarkably, En-1 overexpression rescued these ultrastructural defects, promoting cristae reorganization and shifting the dynamic balance from excessive fission toward fusion (Fig. 5B). To identify the specific mediator of this morphological rescue, we cross-referenced our eight candidates with mitochondrial dynamics regulators in the GeneCards database (https://www.genecards.org/). *PDGFC* emerged as the sole candidate annotated for modulating mitochondrial fission/fusion (Fig. 5C). Bioinformatics analysis identified a conserved En-1 recognition motif within the PDGFC promoter (Fig. 5D, E), and dual-luciferase reporter assays confirmed that En-1 directly binds to this region to potently enhance its transcriptional activity (Fig. 5F).

To delineate the functional contribution of *PDGFC* to En-1-mediated mitochondrial homeostasis, we performed siRNA-mediated knockdown (Fig. 5G–H). We first systematically screened key regulatory proteins involved in fusion (OPA1, MFN1, MFN2) and fission (Drp1, Fis1) in total cellular lysates to identify the specific downstream effectors governed by the En-1/PDGFC axis. Western blot analysis revealed that while several markers were assessed, OPA1 exhibited the most significant and consistent differential expression (Fig. 5G, I). Specifically, En-1 overexpression robustly upregulated OPA1, an effect that was almost entirely abolished upon PDGFC knockdown (OE-En1+si-PDGFC group), whereas other fusion proteins (MFN1/2) and fission-related markers (Drp1, p-Drp1-Ser616, Fis1) remained relatively stable.

To further validate these findings at the organellar level, we employed a differential centrifugation-based approach to isolate highly purified mitochondrial fractions. The efficacy of this isolation was confirmed by a robust VDAC1 signal and a negligible β-actin signal, indicating minimal cytosolic contamination and the high purity of the extracted mitochondria. Consistent with the total protein results, Western blot analysis of these fractions demonstrated that OPA1 levels strictly tracked with En-1 and PDGFC status (Fig. 5J, K). Collectively, these results demonstrate that the En-1/PDGFC axis does not exert a global effect on all mitochondrial proteins, but rather selectively modulates mitochondrial fusion homeostasis by targeting OPA1.

### The transplantation of mitochondria modified to overexpress En-1 enhanced tolerance to SCI/RI both in vivo and in vitro

#### Functional validation and time-dependent uptake of exogenous mitochondria

**Fig. 6:**
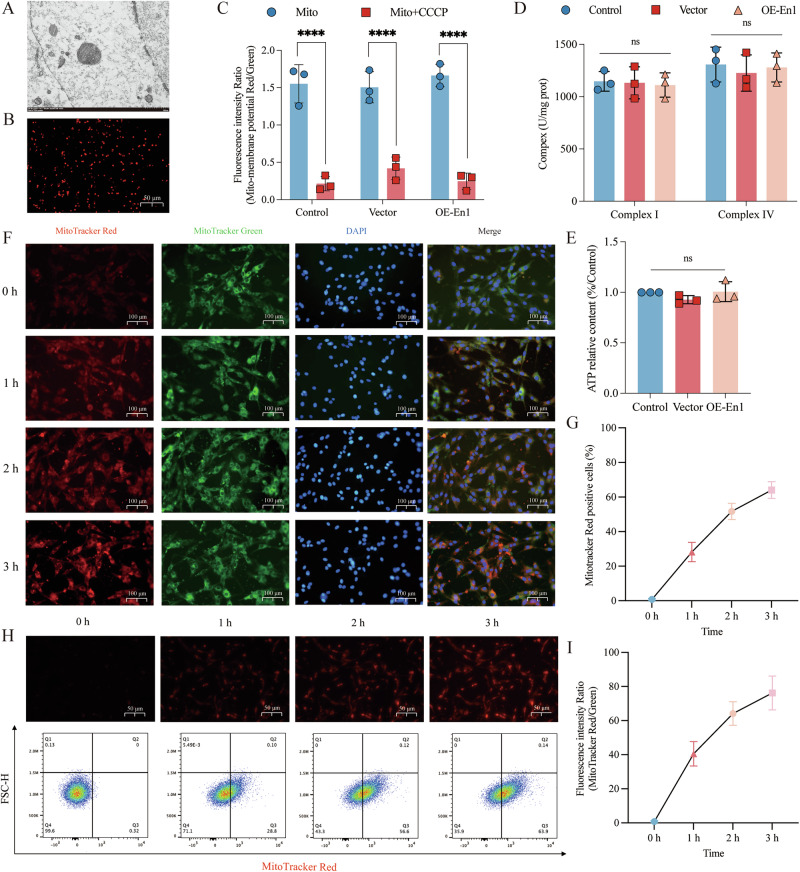
Quality control of exogenous mitochondria and their time-dependent uptake by cells. **A** Transmission electron microscopy of exogenous mitochondria, showing clear inner and outer membranes, numerous and evenly distributed cristae, and intact morphology. Scale bars: 500 nm. **B** MitoTracker Red staining, with numerous mitochondria emitting red fluorescence within the field of view. Scale bars: 50 μm. **C** JC-1 assay for mitochondrial membrane potential in isolated mitochondria vs. CCCP-treated mitochondria. Scale bars: 100 μm. **D** Content of mitochondrial respiratory chain Complex I and IV in isolated mitochondria. **E** ATP content in isolated mitochondria. **F**, **G** MitoTracker Red/Green staining for labeling endogenous and exogenous mitochondria, followed by co-culture experiments, demonstrating time-dependent uptake of exogenous mitochondria by cells. **H**, **I** Fixed-position observations and flow cytometry further confirming the time-dependent uptake of exogenous mitochondria. Scale bars: 50 μm. All data are presented as mean ± SD (*n* = 3) from three independent experiments. Error bars represent SD, and significance was assessed by one-way ANOVA (****P < 0.0001).

High-purity mitochondria were isolated from PC12 cells via standardized centrifugation. TEM verified intact double membranes and dense cristae, confirming minimal structural damage (Fig. 6A). Biological viability was validated by MitoTracker Red and JC-1 assays, the latter showing significantly higher membrane potential than CCCP-treated controls (Fig. 6B, C). Preserved bioenergetic competence was further evidenced by robust ATP production and electron transport chain activities (Fig. 6D, E). These findings confirm that the isolated mitochondria retained the structural and functional integrity requisite for transplantation (MT).

To optimize the transplantation window, exogenous (MitoTracker Red) and endogenous (MitoTracker Green) mitochondria were co-tracked via fluorescence microscopy. We observed a time-dependent internalization pattern, which was further quantified by flow cytometry (Fig. 6F–I). The uptake rate peaked within the first 0–2 hours post-transplantation before reaching a plateau after 3 hours. Consequently, a 2-hour incubation was identified as the optimal duration for subsequent in vitro assays.

#### In vitro validation of the protective effect of “Enhanced” mitochondria

**Fig. 7:**
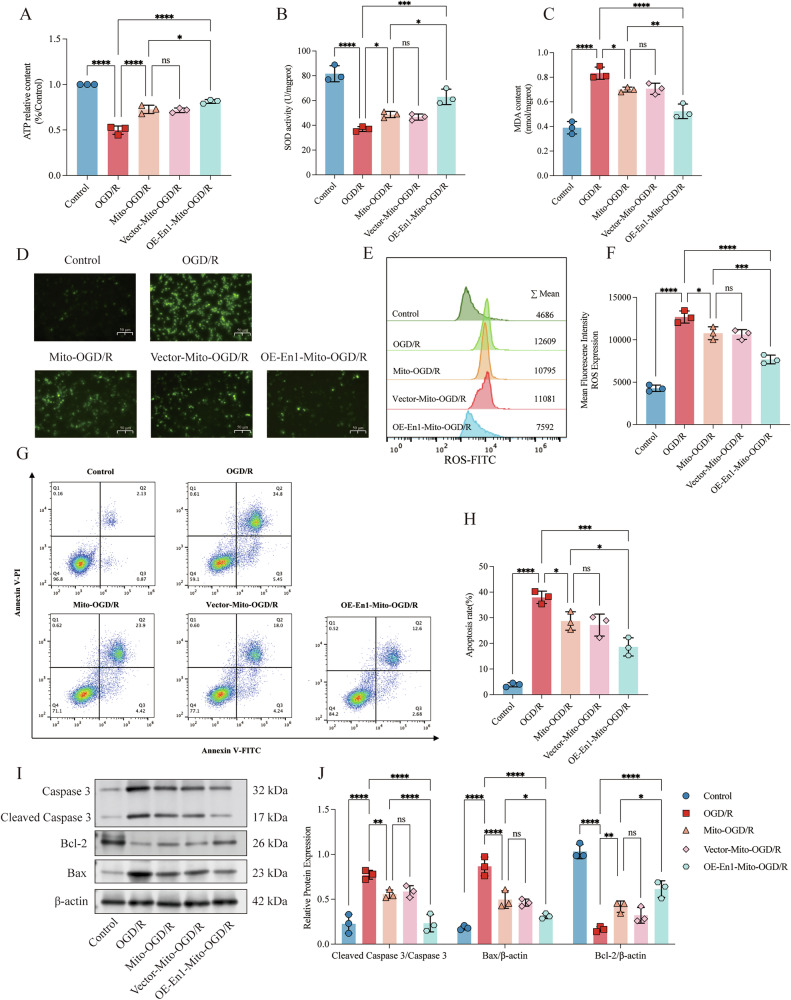
In vitro experiments demonstrating the stronger protective effect of “Enhanced” mitochondria. **A** ATP content measurement. **B** SOD activity assay. **C** MDA content measurement. **D**–**F** Fluorescence images of ROS staining and flow cytometry analysis showing that the ROS levels in the OE-En1-Mito-OGD/R group were significantly lower than those in the Mito-OGD/R group after OGD/R injury. Scale bars: 50 μm. (**G**, **H**) Annexin V flow cytometry analysis to detect cell apoptosis. **I**, **J** Western blot analysis to verify changes in apoptosis-related proteins. All data are presented as mean ± SD (*n* = 3) from three independent experiments. Error bars represent SD, and significance was assessed by one-way ANOVA (*P < 0.05, **P < 0.01, ***P < 0.001, ****P < 0.0001).

To validate the superior cytoprotective efficacy of En-1-modified mitochondria, we compared three exogenous groups: normal mitochondria (Mito), vector-transfected mitochondria (Vector-Mito), and En-1-overexpressing mitochondria (OE-En1-Mito). Following a 2-hour pre-incubation with 100 μg/mL mitochondria, PC12 cells were subjected to OGD/R injury. Metabolic and oxidative stress profiling revealed that the OE-En1-Mito group exhibited significantly enhanced bioenergetic and antioxidant capacity compared to the Mito group. Specifically, ATP content and SOD activity were markedly elevated (Fig. 7A, B), while MDA and ROS levels were significantly suppressed (Fig. 7C–F).

Flow cytometric analysis further demonstrated that OE-En1-Mito transplantation significantly attenuated the apoptosis rate compared to the Mito-OGD/R group (*P* < 0.05; Fig. 7G, H). This neuroprotective effect was corroborated by Western blot analysis, which showed a reciprocal modulation of apoptotic markers: a robust upregulation of anti-apoptotic Bcl-2 alongside a significant reduction in pro-apoptotic Bax and cleaved caspase-3 (Fig. 7I, J). Collectively, these findings substantiate that En-1 overexpression optimizes the functional potency of exogenous mitochondria, providing superior defense against oxidative stress-induced cell death.

#### “Enhanced” mitochondria provided strong protective effects in rat SCI/RI Models

**Fig. 8:**
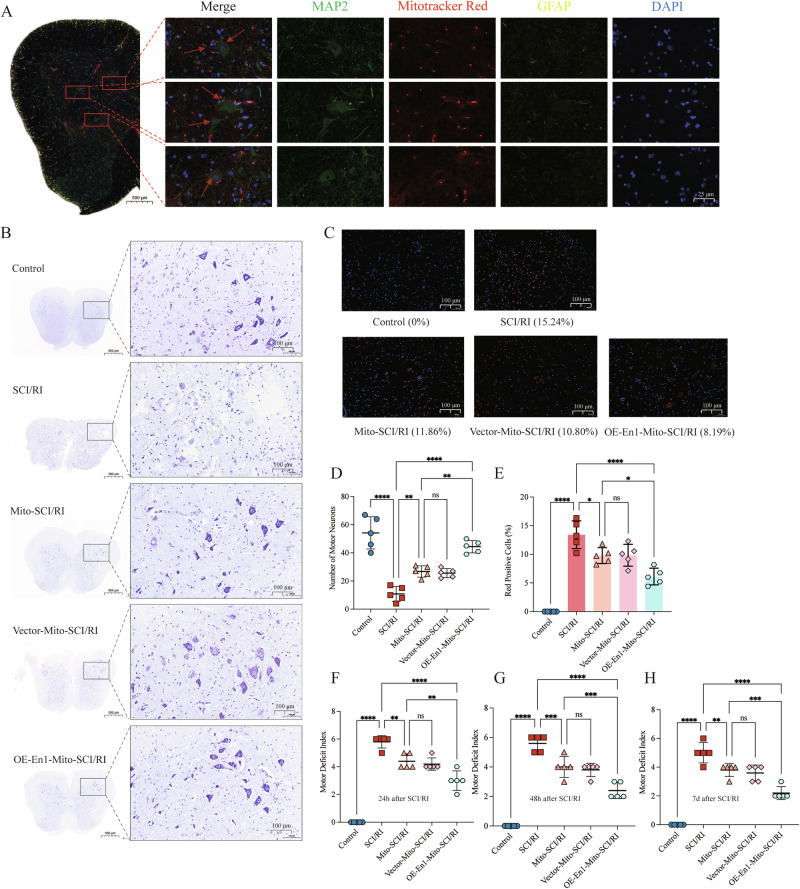
In vivo experiments demonstrating the stronger protective effect of “Enhanced” mitochondria. **A** Co-localization of rat spinal cord neurons with exogenous mitochondria (indicated by red arrows). Scale bars: 500 μm (whole spinal cord) and 25 μm (magnified views). **B** Nissl staining. Scale bars: 500 μm (whole spinal cord) and 100 μm (magnified views). **C** TUNEL staining. Scale bars: 100 μm. **D** Nissl staining analysis. **E** TUNEL staining analysis. **F**–**H** MDI scores. Data are presented as mean ± SD. For the nonparametric data (Nissl staining analysis and MDI scores), statistical significance was determined by the Mann-Whitney U test. For TUNEL staining analysis, statistical significance was determined by one-way ANOVA. A *P* value < 0.01 was considered statistically significant. n = 5 per group in all experiments. (*P < 0.05, **P < 0.01, ***P < 0.001, ****P < 0.0001).

Before evaluating therapeutic outcomes, we confirmed the internalization efficiency of PC12-derived mitochondria by rat spinal cord neurons. Exogenous mitochondria, labeled with MitoTracker Red, were delivered via intrathecal injection. Immunofluorescence analysis 24 hours post-injection revealed prominent co-localization of red mitochondrial signals with MAP2-positive neurons, whereas minimal co-localization was observed with GFAP-positive astrocytes (Fig. 8A). These results demonstrate the cell-type-specific uptake of exogenous mitochondria by spinal cord neurons, establishing a critical foundation for evaluating their neuroprotective effects.

Nissl staining (Fig. 8B) was employed to assess structural neuronal integrity. The SCI/RI group exhibited severe Nissl body dissolution and widespread tissue vacuolization. While Mito-SCI/RI and Vector-Mito-SCI/RI treatments partially mitigated this damage, the OE-En1-Mito-SCI/RI group demonstrated the most robust preservation of neuronal architecture. In this group, neurons retained their typical polygonal morphology with centrally located nuclei and significantly higher Nissl body counts compared to other treatment groups (*n* = 5, *P* < 0.01; Fig. 8D). Consistently, TUNEL staining (Fig. 8C, E) confirmed that the OE-En1-Mito-SCI/RI group had the lowest apoptosis rate (*n* = 5, *P* < 0.05), highlighting its superior anti-apoptotic capacity in vivo.

Finally, we objectively assessed hindlimb motor recovery using the Motor Deficit Index (MDI) at 24 h, 48 h, and 7 days post-surgery. The OE-En1-Mito-SCI/RI group displayed significantly lower MDI scores compared to all other treatment groups across all time points (*n* = 5, *P* < 0.05; Fig. 8F–H). These behavioral findings further substantiate that En-1-modified mitochondrial transplantation significantly enhances spinal cord ischemic tolerance and accelerates functional motor recovery.

#### PDGFC knockdown abrogates En1-mediated mitochondrial fusion enhancement

**Fig. 9:**
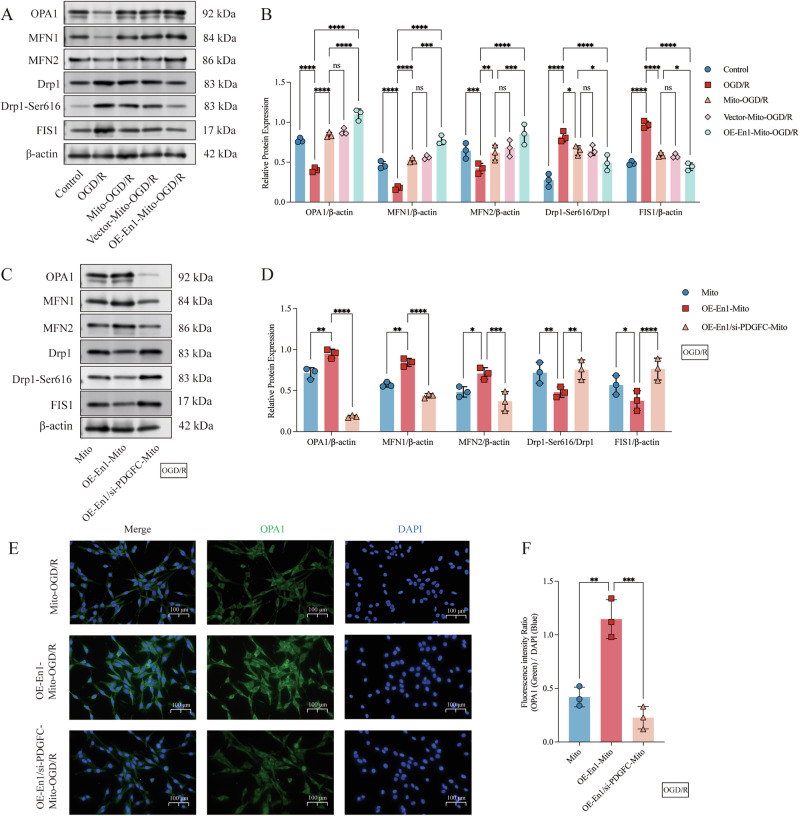
Verification of PDGFC-Mediated Enhancement of Mitochondrial Fusion Function After Mitochondrial Transplantation. **A**, **B** Western blot analysis to verify the expression changes of mitochondrial fission/fusion proteins after mitochondrial transplantation and OGD/R injury. **C**, **D** Western blot analysis to verify the expression changes of mitochondrial fission/fusion proteins after mitochondrial transplantation and OGD/R injury following PDGFC interference. **E**, **F** Immunofluorescence and analysis of OPA1 protein. Scale bars: 100 μm. All data are presented as mean ± SD (*n* = 3) from three independent experiments. Error bars represent SD, and significance was assessed by one-way ANOVA (*P < 0.05, **P < 0.01, ***P < 0.001, ****P < 0.0001).

To determine whether En-1-modified mitochondria maintain their superior fusion capacity after internalization, we assessed the regulatory effects of En-1 on mitochondrial dynamics following MT. Western blot analysis showed that the OE-En1-Mito group exhibited a significant shift toward mitochondrial fusion, characterized by the upregulation of fusion-related proteins and a concomitant reduction in fission markers (Fig. 9A, B). These data reinforce that En-1 pre-conditioning augments the intrinsic fusion capacity of donor mitochondria via the PDGFC pathway, thereby enhancing their neuroprotective efficacy in recipient cells.

To further investigate the functional requirement of PDGFC in this mechanism, we utilized exogenous mitochondria harvested from cells with PDGFC knockdown. Following transplantation into PC12 cells under OGD/R conditions, Western blot analysis demonstrated that the expression levels of key fusion proteins were markedly diminished in the absence of donor PDGFC (Fig. 9C, D). Crucially, focused immunofluorescence analysis of OPA1 confirmed this significant reduction at the morphological level (Fig. 9E, F). Given that OPA1 exhibited the most pronounced differential expression in our initial protein screening, these results support the notion that the En-1-mediated enhancement of mitochondrial fusion is closely linked to the PDGFC/OPA1 axis.

## Discussion

This study expands the functional repertoire of En-1, revealing for the first time its pivotal protective role in ischemia-reperfusion injury. Through integrated CUT&Tag and RNA-seq analysis, we identified PDGFC as a direct transcriptional target of En-1, established via specific promoter binding. This discovery defines a novel En-1/PDGFC signaling axis essential for neural protection. Notably, MT experiments revealed that En-1-modified “enhanced” mitochondria confer superior protection in SCI/RI, a process driven by the targeted upregulation of the fusion protein OPA1 within the donor organelles. These findings refine the mechanistic understanding of En-1-mediated neuroprotection and offer promising therapeutic targets for spinal cord injury.

While En-1 is essential for neural development and maintenance^38,39^, its regulatory role in pathological states like ischemia-reperfusion remains poorly understood. En-1 is highly expressed under hypoxia and protects against oxidative stress^25^, features highly relevant to SCI/RI. In this study, hypoxic preconditioning^36,40^ increased En-1 expression in PC12 cells, significantly improving viability after OGD/R injury. Importantly, we report for the first time that En-1 is significantly downregulated in acute SCI/RI models—a finding corroborated in OGD/R-treated PC12 cells. This dynamic expression pattern—early downregulation followed by potential late-phase upregulation—suggests that En-1 contributes differentially to injury progression and endogenous repair. This fluctuation directly contrasts with the sustained activation of HIF-1α^41,42^, indicating that En-1 responds to ischemic stress via specialized, non-HIF-dependent pathways.

Mechanistically, En-1 exerts complex regulatory functions across multiple signaling networks. While En-1 modulates apoptosis, MYC, and MAPK signaling in diverse contexts^43,44^, its neuroprotective role is closely tied to mitochondrial integrity. For instance, in dopaminergic neurons, En-1 regulates mitochondrial signaling to activate survival pathways^28,45^. Consistently, our study demonstrates that En-1 overexpression effectively maintains mitochondrial membrane potential, reduces ROS accumulation, and inhibits apoptosis following OGD/R injury. These findings confirm that En-1 exerts a central neuroprotective role by preserving neuronal mitochondrial integrity, providing a deeper mechanistic understanding of its therapeutic potential and identifying novel targets for SCI/RI intervention.

Under OGD/R stress conditions, the downstream regulatory network of En-1 undergoes substantial reprogramming, with its target genes transitioning from development-related genes (e.g., *WNT5A*^46,47^, *PAX6*^48^) to those involved in metabolism and stress responses (e.g., *LINE1*^49^, *COL22A1*^50^). This environment-dependent DNA binding preference identifies En-1 as a “stress-responsive” transcriptional regulator. Through integrated CUT&Tag and RNA-seq analysis, we revealed for the first time that En-1 directly binds to the *PDGFC* promoter, with dual-luciferase assays confirming that En-1 positively regulates its transcriptional activity. This systematic shift in cellular processes underscores the adaptive role of En-1 in response to ischemic insult.

PDGFC, a potent growth factor signaling through PDGFRα/αβ receptors, has emerged as a key regulator of mitochondrial homeostasis. While PDGFC is known to inhibit superoxide production via SOD2 and *KEAP1* modulation^51^, its influence on mitochondrial morphology remains less defined. Recent evidence suggests that PDGFC reverses DRP1-mediated mitochondrial fission induced by high-glucose stress^52,53^, though the underlying mechanism has been elusive. In this study, we demonstrate that PDGFC improves mitochondrial dynamics by specifically upregulating the fusion protein OPA1. This mechanism was rigorously validated through combined En-1 overexpression and PDGFC interference experiments, identifying OPA1 as a critical downstream effector of the En-1/PDGFC axis.

In this study, En-1 overexpression significantly elevated OPA1 levels, leading to a 17.7% increase in mitochondrial membrane potential, a 51% reduction in ROS generation, and sustained ATP synthesis. These metabolic improvements directly correlated with a 21.5% decrease in apoptosis. As a master regulator of mitochondrial inner membrane fusion, OPA1 upregulation not only promotes mitochondrial network reorganization^54,55^ but also stabilizes cristae structure to inhibit cytochrome c release^56,57^. This structural preservation likely serves as the mechanistic basis for En-1-mediated anti-apoptosis. Ultimately, the elucidation of the En-1/PDGFC axis provides a novel theoretical framework for the transcriptional regulation of mitochondrial homeostasis.

While gene therapy holds immense potential, direct genomic manipulation carries inherent risks, including genomic instability, adverse immune responses, and long-term safety concerns such as oncogenesis^58,59^. In contrast, acellular therapy^60,61^ represents a safer, precision-driven alternative. By targeting the repair or replacement of damaged cellular components rather than altering the genome, this emerging strategy facilitates the restoration of specific cellular functions while bypassing the ethical and safety complexities of direct genetic modification.

MT^62^ has emerged as a pioneering acellular therapy for restoring cellular bioenergetics in diverse tissues. In SCI/RI, mitochondrial dysfunction drives metabolic collapse and triggers neuronal apoptosis^63,64^. While strategies such as photobiomodulation-induced transfer^65^ or direct transplantation of isolated mitochondria^33^ have shown efficacy in reducing oxidative stress and improving motor function, “passive” transplantation may be insufficient for optimal repair. Evidence suggests that the therapeutic potency of MT is highly dependent on donor mitochondrial “fitness.” For instance, pre-activating ALDH2 in donor mitochondria with Alda-1 significantly enhances their survival and anti-apoptotic capacity in cardiomyocytes^20^. This indicates that the clinical success of MT relies not only on mitochondrial delivery but also on targeted modifications or pathway activation within the organelles prior to transplantation.

We propose a novel “transcription factor pre-modification” strategy to enhance mitochondrial function, distinguishing our approach from conventional MT or enzyme activation methods. Mitochondria overexpressing En-1 (OE-En1-Mito) exhibited superior therapeutic efficacy post-transplantation: recipient cells showed a 1.5-fold increase in SOD activity and a 38% reduction in MDA levels. In vivo, intrathecal injection of OE-En1-Mito significantly improved motor function (MDI) scores and increased Nissl body retention by ~30% at 7 days post-surgery. Mechanistically, this enhanced protection relies on the En-1/PDGFC axis, as PDGFC knockdown in donor mitochondria abolished these benefits, confirming its critical role in boosting fusion capacity. This strategy maximizes the protective potential of exogenous mitochondria, offering a new paradigm for optimizing MT therapy.

Biologically, the present study contributes to the understanding of En-1 function, expanding its regulatory scope from developmental processes to protection against ischemic injuries. Moreover, the study establishes a novel neuroprotective pathway, En-1/PDGFC, which offers a new perspective on the role of PDGF family molecules in organelle remodeling. Notably, this pathway provides a theoretical foundation for clinical translation. For instance, in cases involving a high risk of SCI/RI, such as vascular surgery or organ transplantation, the local injection of En-1 agonists or PDGFC nanoparticles could serve as a preventive therapeutic approach. Furthermore, the preparation of En-1-modified mitochondria from patients’ autologous cells may offer personalized treatment options for post-injury repair. Furthermore, the conservation of En-1 binding sites in human ChIP-seq data^66^ indicates its potential for clinical translation, although consideration should be given to the variations in En-1 expression patterns across species. While mouse En-1 primarily contributes to the regulation of dopaminergic neurons in the midbrain^67,68^, the dynamic expression of rat En-1 in SCI observed in this study suggests that its function in the adult nervous system may be tissue-specific.

## Limitation

Despite these findings, several limitations warrant consideration. First, the precise molecular link between PDGFC and OPA1 remains to be fully elucidated; future studies employing gene editing and high-throughput proteomics are needed to map the broader interaction network of this axis. Second, while a specific vehicle control for the mitochondrial storage buffer was not included, the physiological compatibility of the buffer and the significantly superior efficacy of both unmodified and En-1-modified mitochondria relative to the model group ensure that the observed therapeutic benefits are distinguishable from background noise. Third, although the transplantation timing was established, the optimal dosage and spatial distribution of mitochondria require further refinement through dose-gradient experiments and real-time in vivo imaging. Finally, the use of PC12-derived mitochondria poses potential risks of heterologous DNA or immunogenicity. Future research should prioritize long-term safety assessments and the development of autologous mitochondrial transplantation strategies to facilitate safe and effective clinical translation.

## Conclusion

In summary, this study provides systematic evidence identifying En-1 as a pivotal neuroprotective transcription factor in SCI/RI. By establishing the En-1/PDGFC/OPA1 axis, we reveal a novel molecular mechanism through which transcriptional reprogramming governs mitochondrial homeostasis and neuronal survival. Furthermore, our proposed “transcription factor pre-modification” strategy significantly enhances the therapeutic potency of mitochondrial transplantation, offering a transformative theoretical framework and promising intervention targets for the clinical management of ischemic spinal cord injury.

## Supplementary information

Supplementary Information

## Data Availability

All high-throughput sequencing data original to this study have been deposited in the National Center for Biotechnology Information (NCBI) Gene Expression Omnibus (GEO). These data can be accessed through the following series accession numbers: GSE322512, GSE322513, and GSE322514. Unedited original blot gel images (Supplementary Figs. 1–6) and FACS gating strategy images (Supplementary Figs. 7–10) are provided in the Supplementary Methods section of the Supplementary Information file. Numerical source data underlying graphs in the manuscript can be found in the Supplementary Data section of the Supplementary Information file.
