# Supplementary material for: Engrailed-1 potentiates mitochondrial transplant neuroprotection in spinal cord ischemia-reperfusion injury
Source: Commun Biol. 2026 May 7;9:1102. doi: 10.1038/s42003-026-10171-6 (PMC13476436; doi:10.1038/s42003-026-10171-6)
Supplement: Supplementary file 1 [file 42003_2026_10171_MOESM1_ESM.pdf]

Supplementary Methods

Supplementary Figure 1: Unedited Blot Gel Images in Figure 1.

Supplementary Figure 2: Unedited Blot Gel Images in Figure 2.

Supplementary Figure 3: Unedited Blot Gel Images in Figure 3.

Supplementary Figure 4: Unedited Blot Gel Images in Figure 5.

Supplementary Figure 5: Unedited Blot Gel Images in Figure 7.

Supplementary Figure 6: Unedited Blot Gel Images in Figure 9.

Supplementary Figure 7: FACS Gating Strategy images in Figure 2.

Supplementary Figure 8: FACS Gating Strategy images in Figure 3.

Supplementary Figure 9: FACS Gating Strategy images in Figure 6.

Supplementary Figure 10: FACS Gating Strategy images in Figure 7.

Supplementary Figure 1: Unedited Blot Gel Images in Figure 1.

Figure 1E

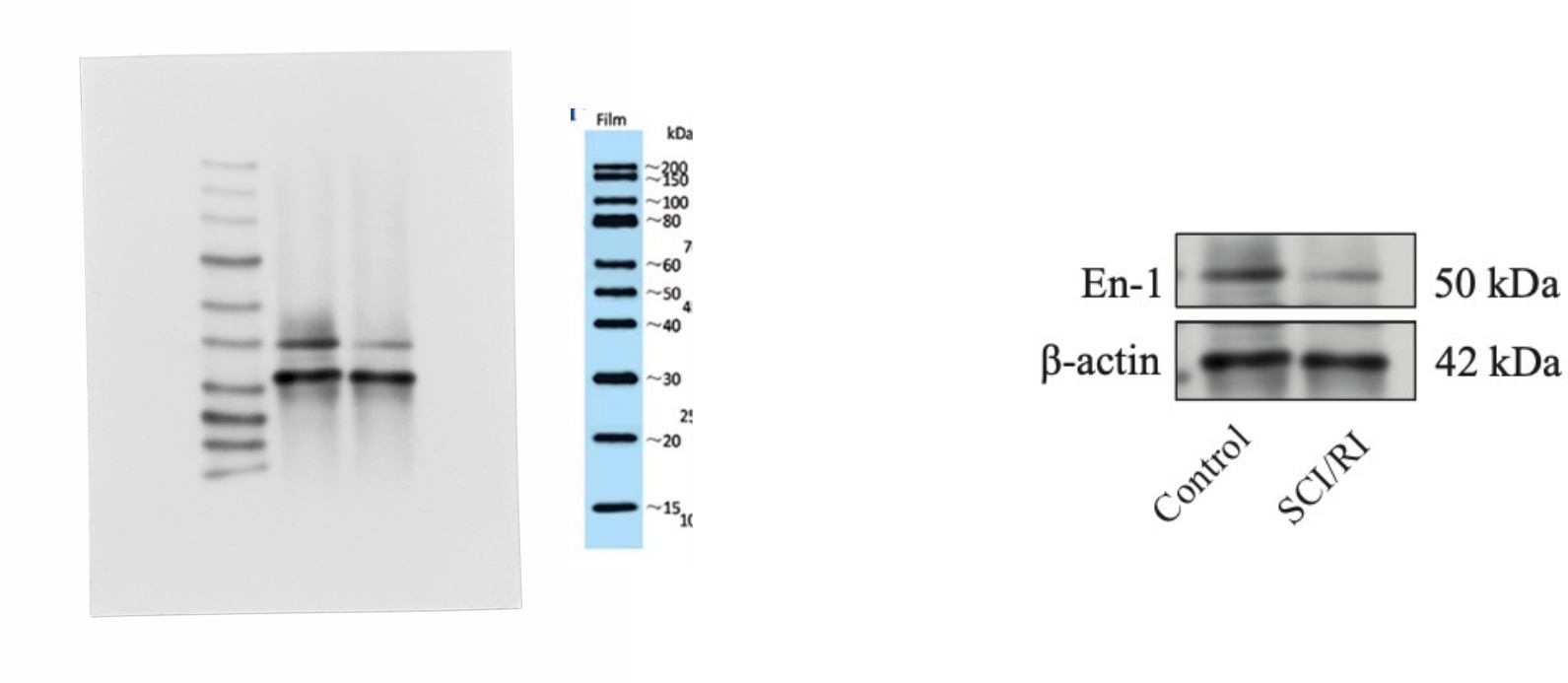

Figure 1H

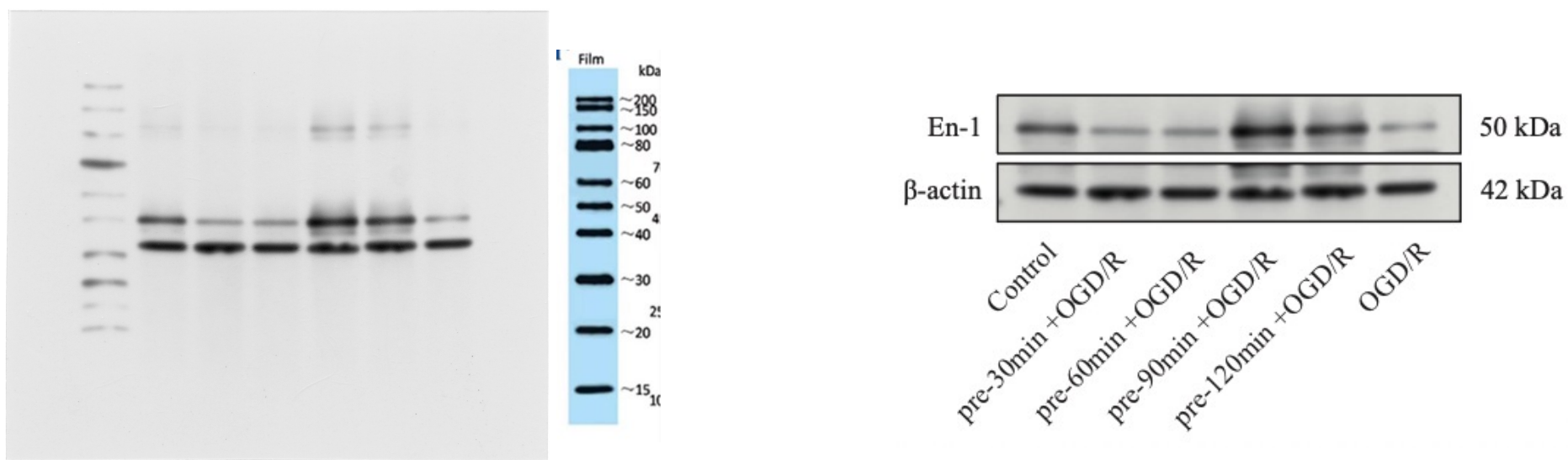

Supplementary Figure 2: Unedited Blot Gel Images in Figure 2.

Figure 2B

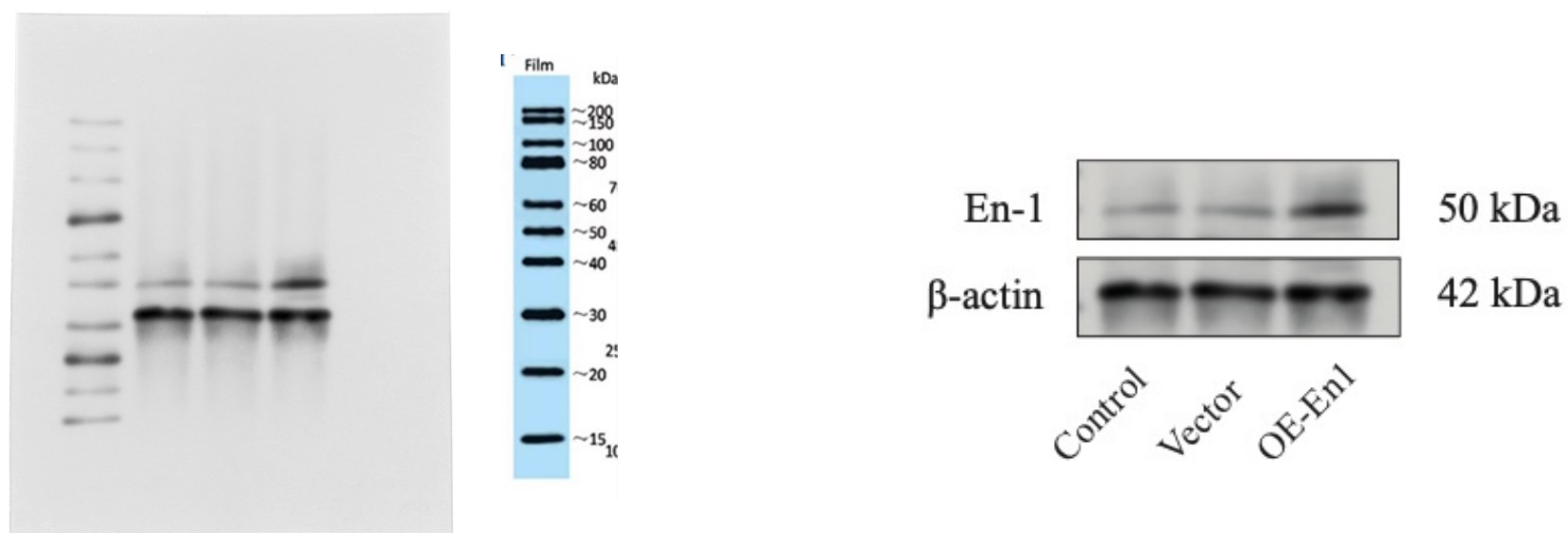

Supplementary Figure 3: Unedited Blot Gel Images in Figure 3.

Figure 3C

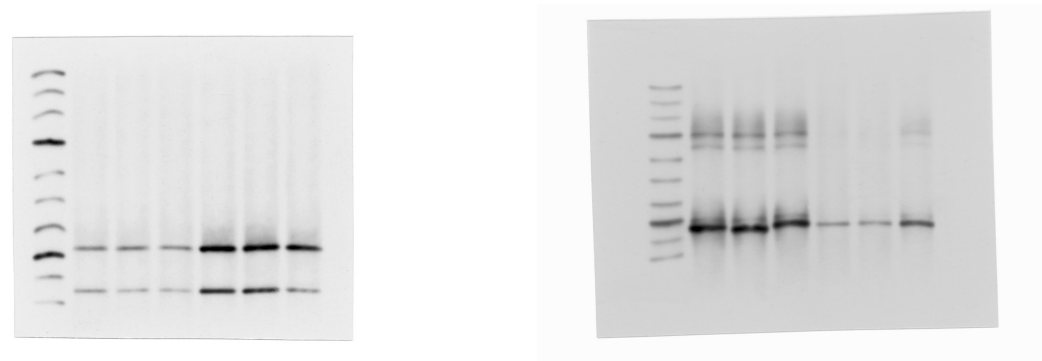

Total-caspase3 32 kDa
Cleaved Caspase 3/Caspase 3 17 kDa

Bcl-2 26 kDa

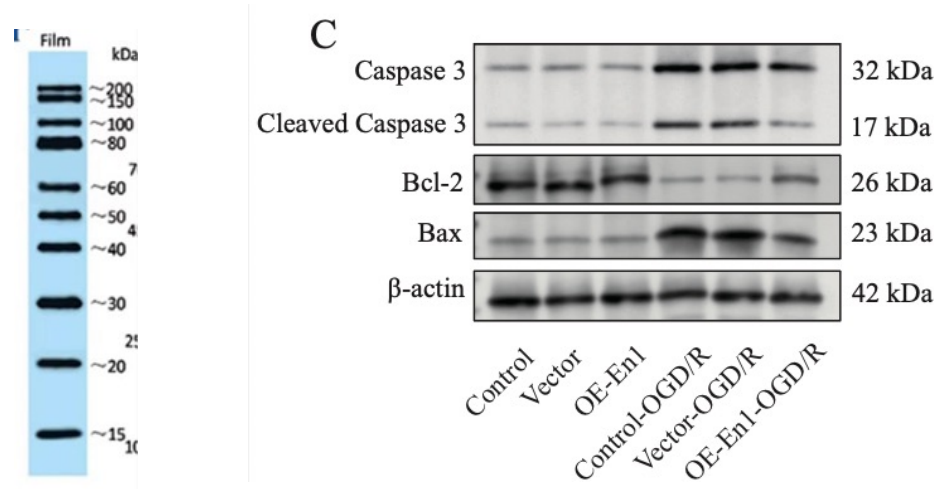

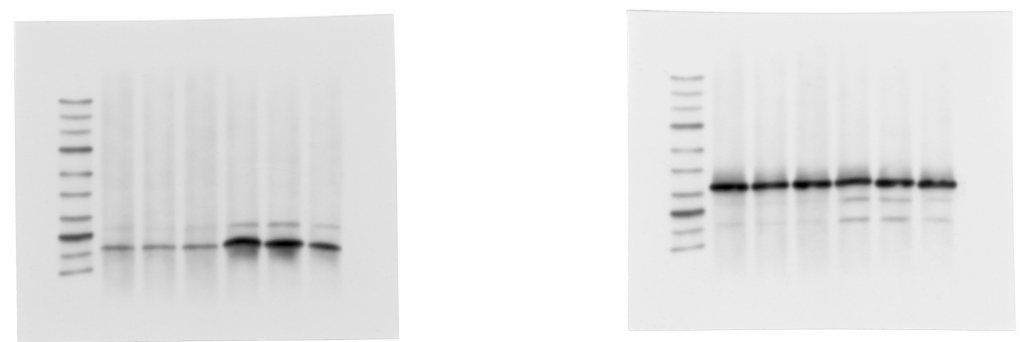

Bax 23 kDa

β-actin 42 kDa

Supplementary Figure 4: Unedited Blot Gel Images in Figure 5.

Figure 5G

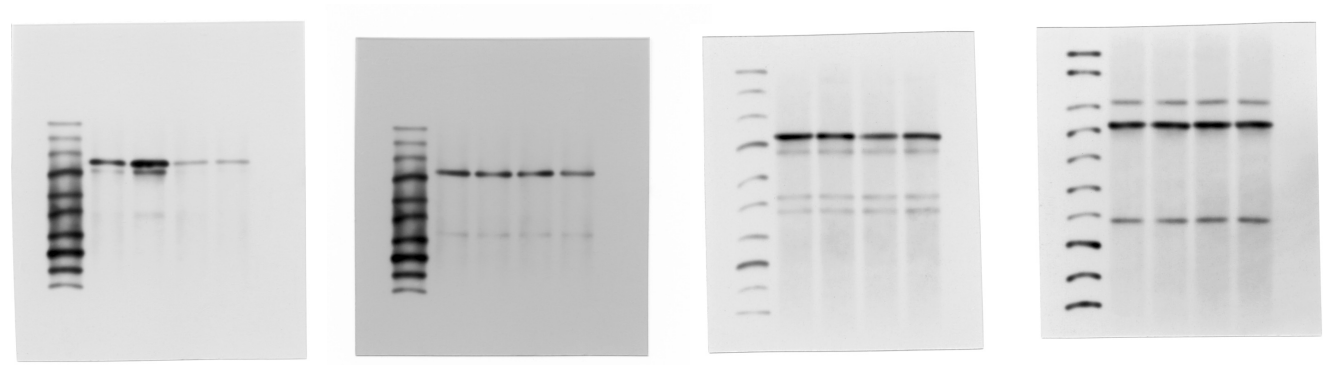

OPA1 92 kDa

MFN1 84 kDa

MFN2 86 kDa

Drp1 83 kDa

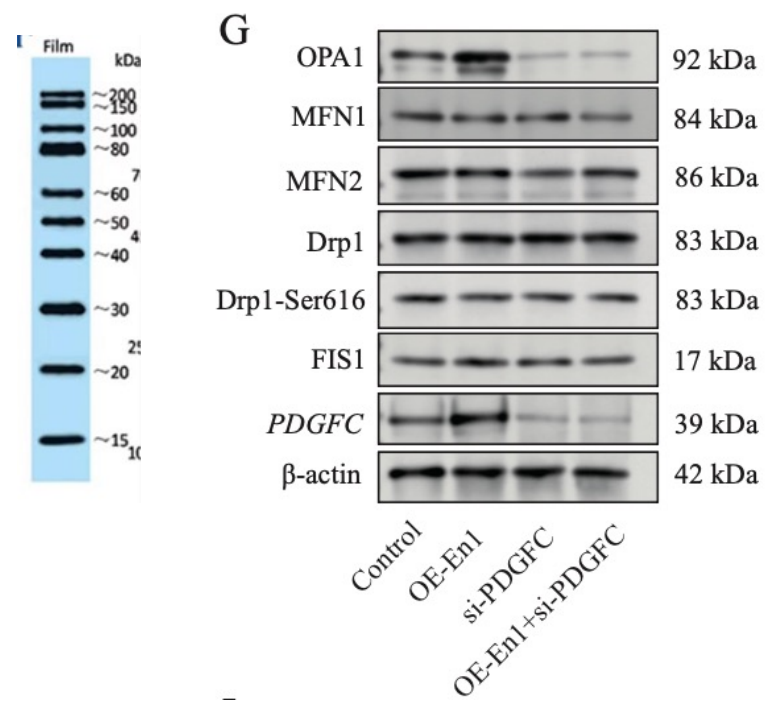

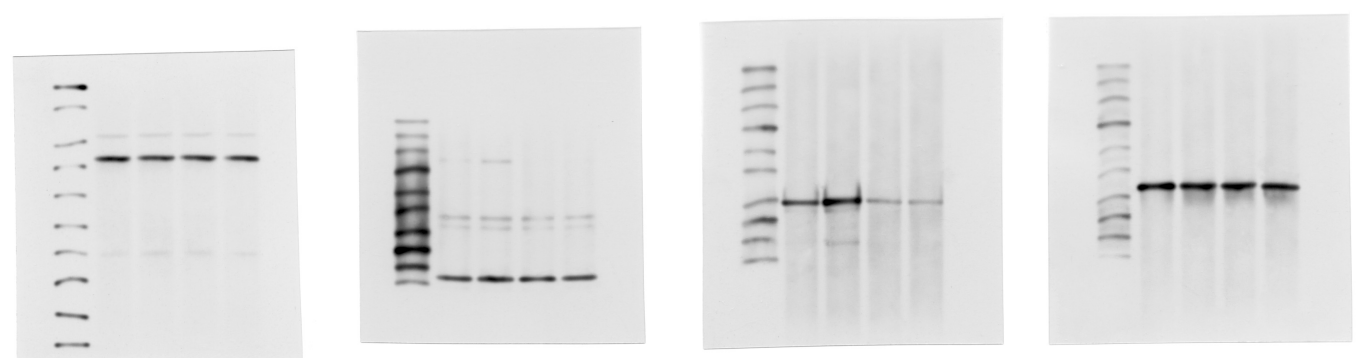

Drp1 Ser616 83 kDa

FIS1 17 kDa

PDGFC 39 kDa

β-actin 42 kDa

Figure 5J

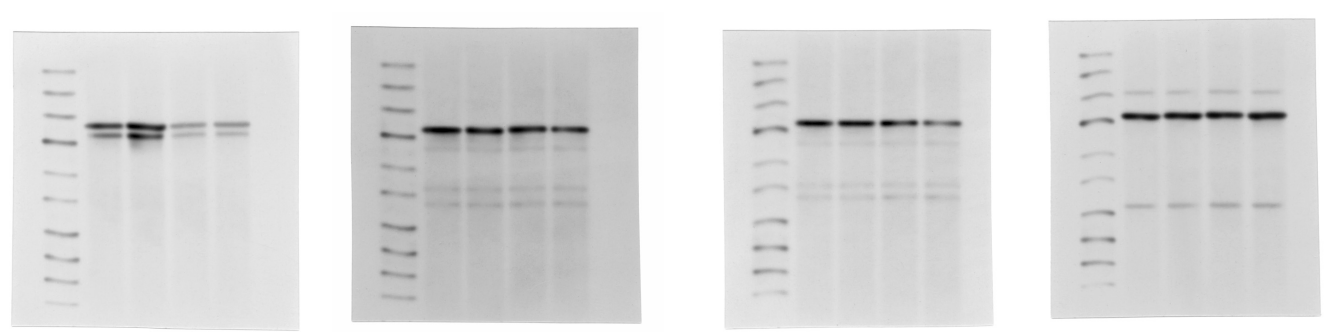

OPA1 92 kDa

MFN1 84 kDa

MFN2 86 kDa

Drp1 83 kDa

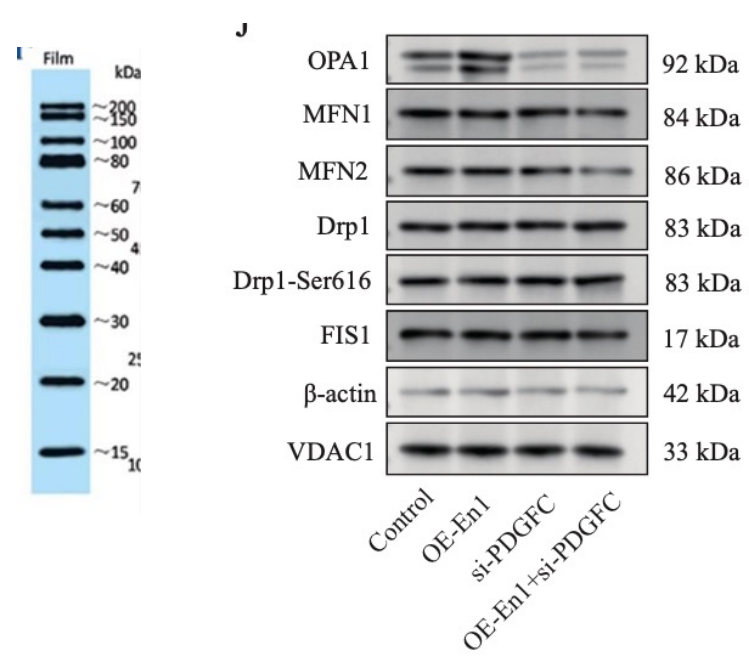

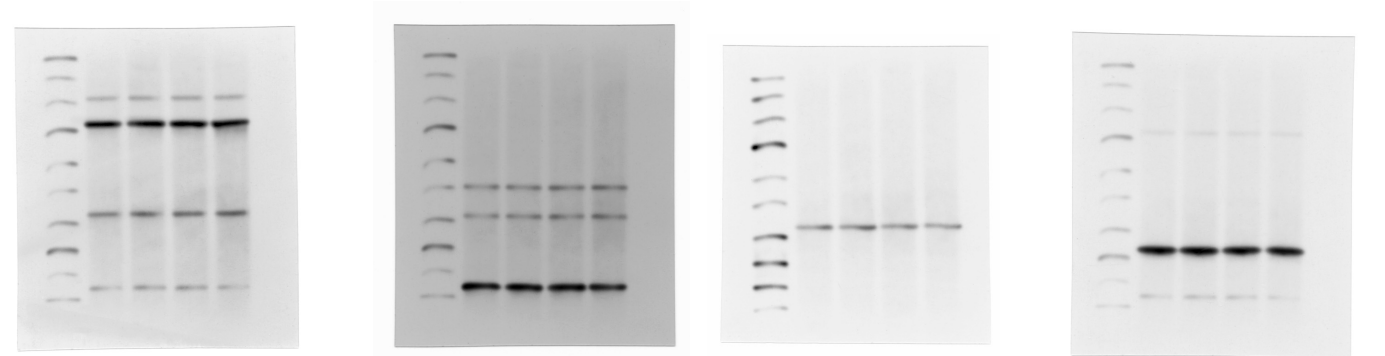

Drp1 Ser616 83 kDa

FIS1 17 kDa

β-actin 42 kDa

VDAC1 33 kDa

Supplementary Figure 5: Unedited Blot Gel Images in Figure 7.

Figure 7I

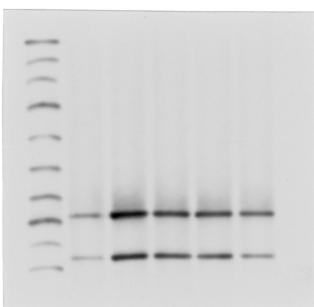

Total-caspase3 32 kDa
Cleaved Caspase 3/Caspase 3   17 kDa

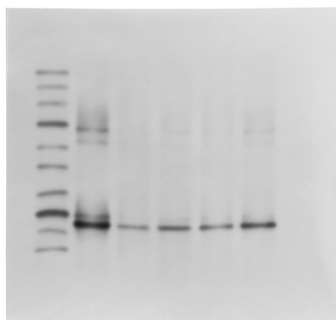

Bcl-2 26 kDa

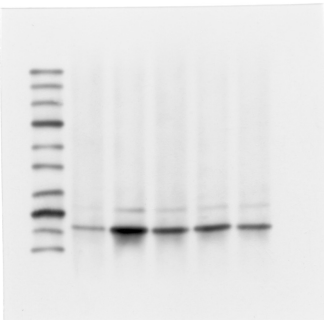

Bax 23 kDa

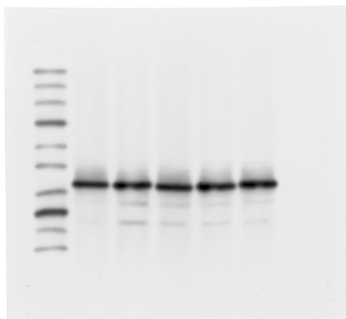

β-actin 42 kDa

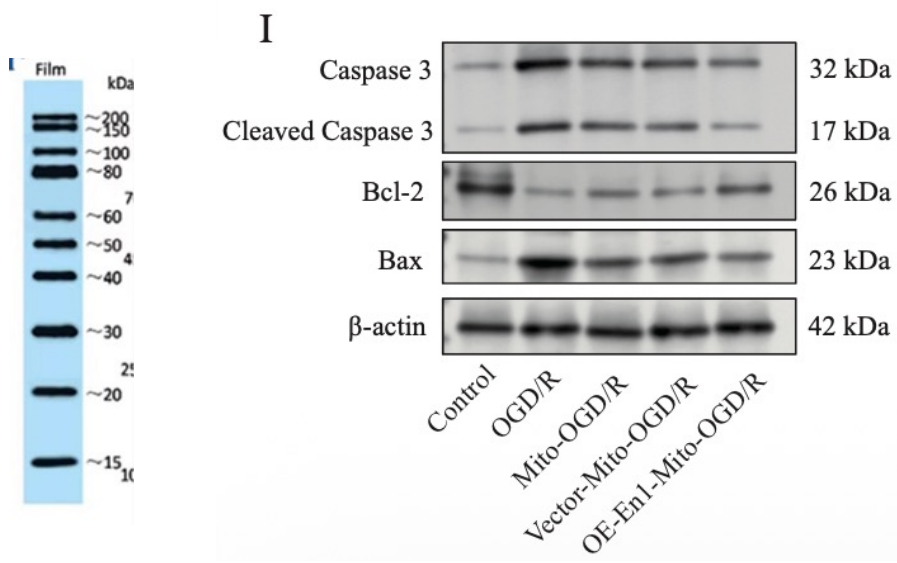

Supplementary Figure 6: Unedited Blot Gel Images in Figure 9.

Figure 9A

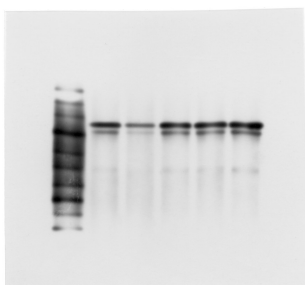

OPA1 92 kDa

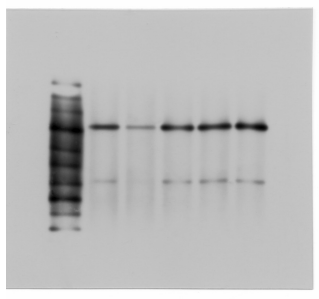

MFN1 84 kDa

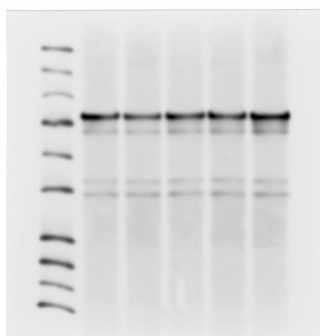

MFN2 86 kDa

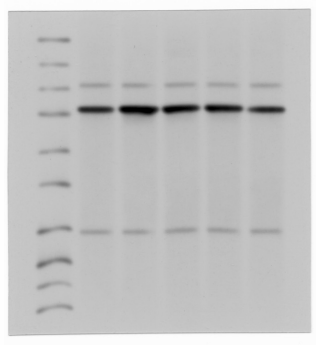

Drp1 83 kDa

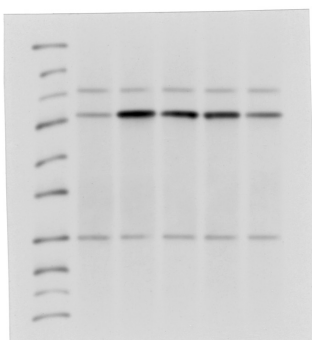

Drp1 Ser616 83 kDa

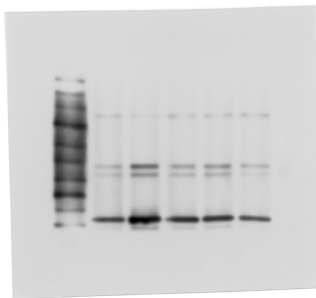

FIS1 17 kDa

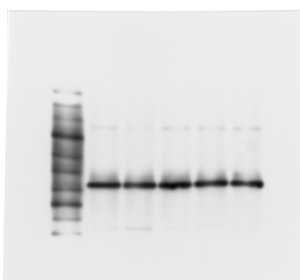

β-actin 42 kDa

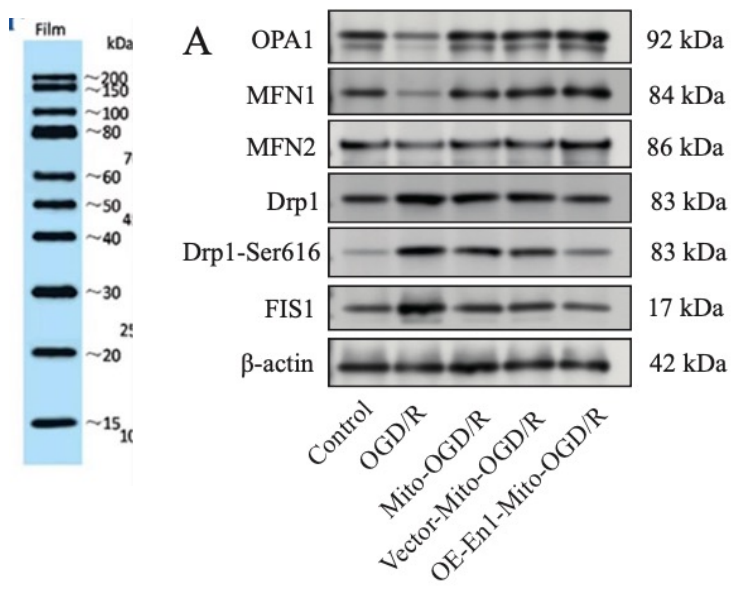

Figure 9C

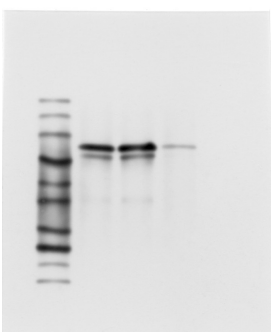

OPA1 92 kDa

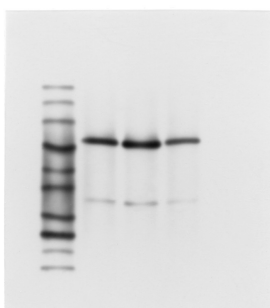

MFN1 84 kDa

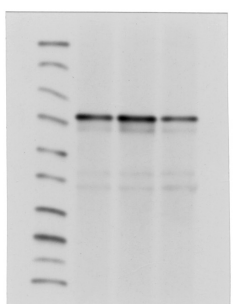

MFN2 86 kDa

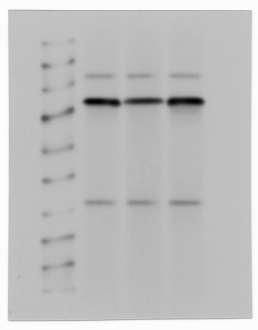

Drp1 83 kDa

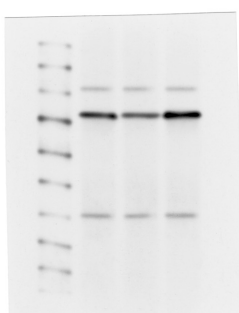

Drp1 Ser616 83 kDa

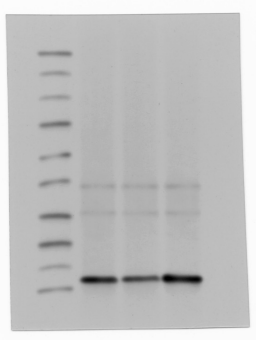

FIS1 17 kDa

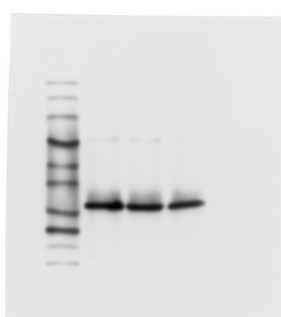

β-actin 42 kDa

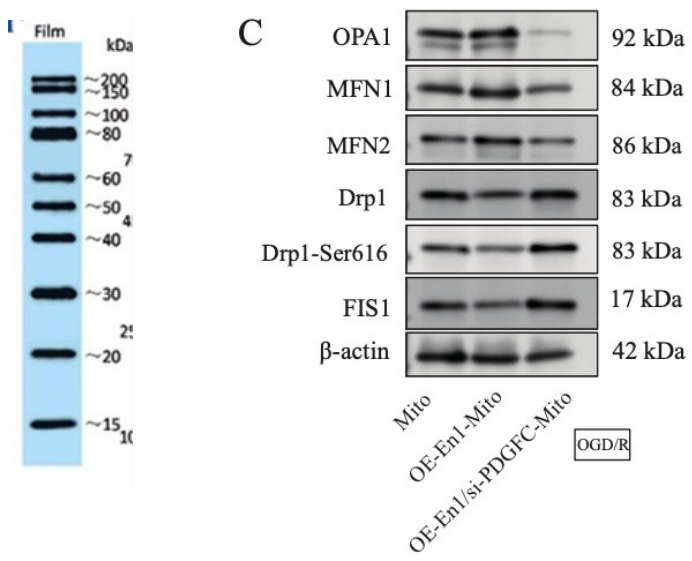

Supplementary Figure 7: FACS Gating Strategy images in Figure 2.

Figure 2G

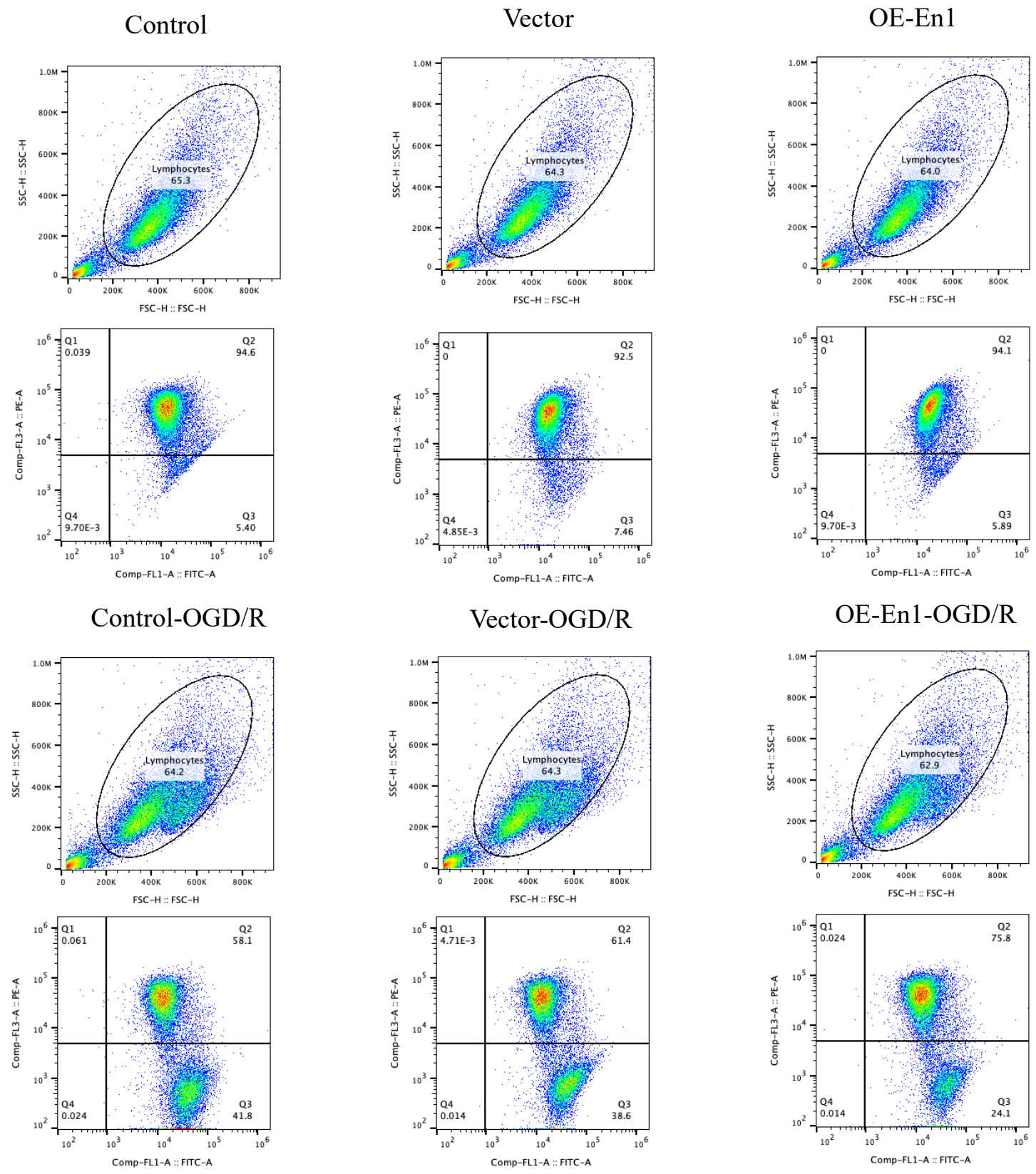

Figure 2K

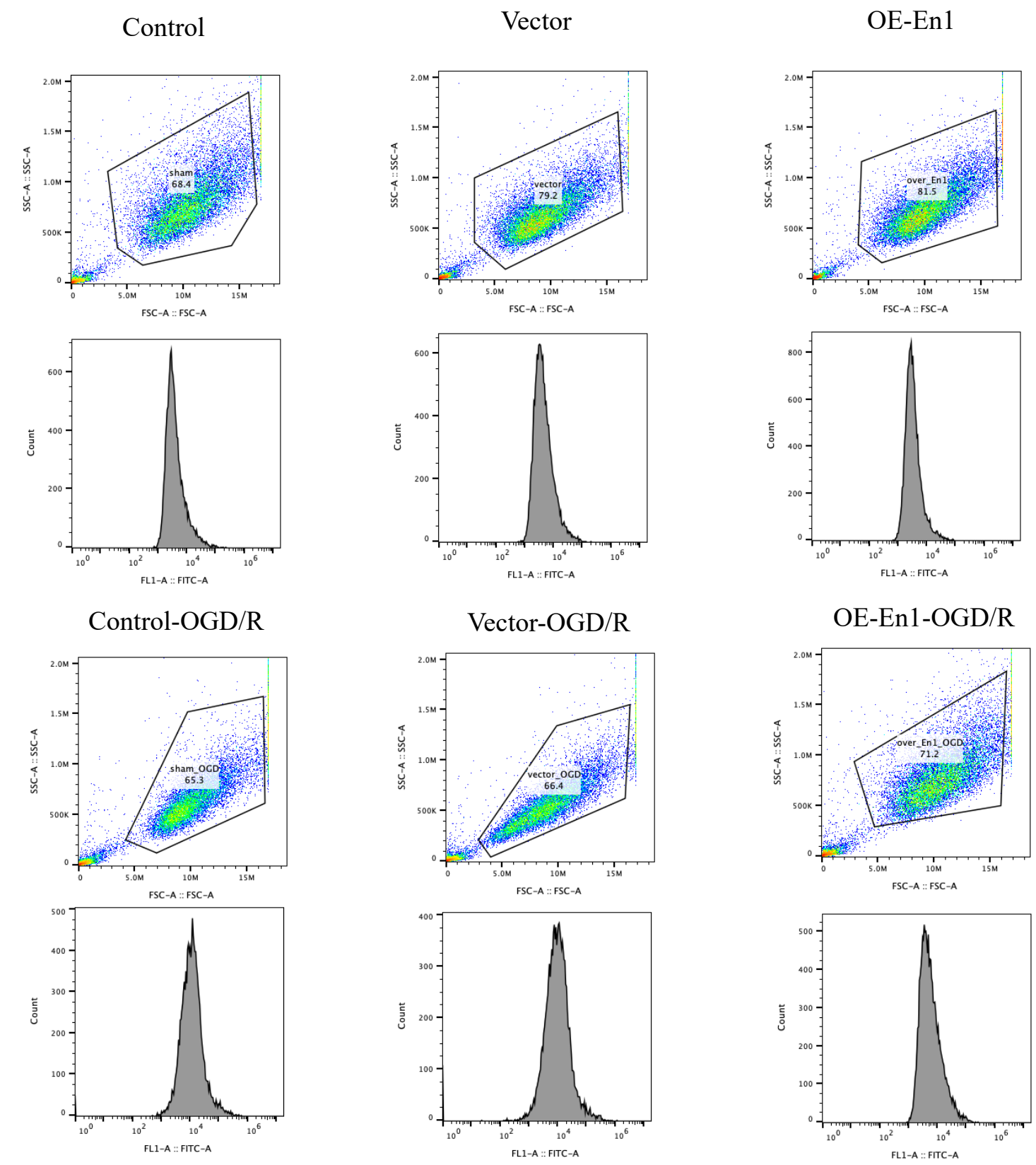

Supplementary Figure 8: FACS Gating Strategy images in Figure 3.

Figure 3A

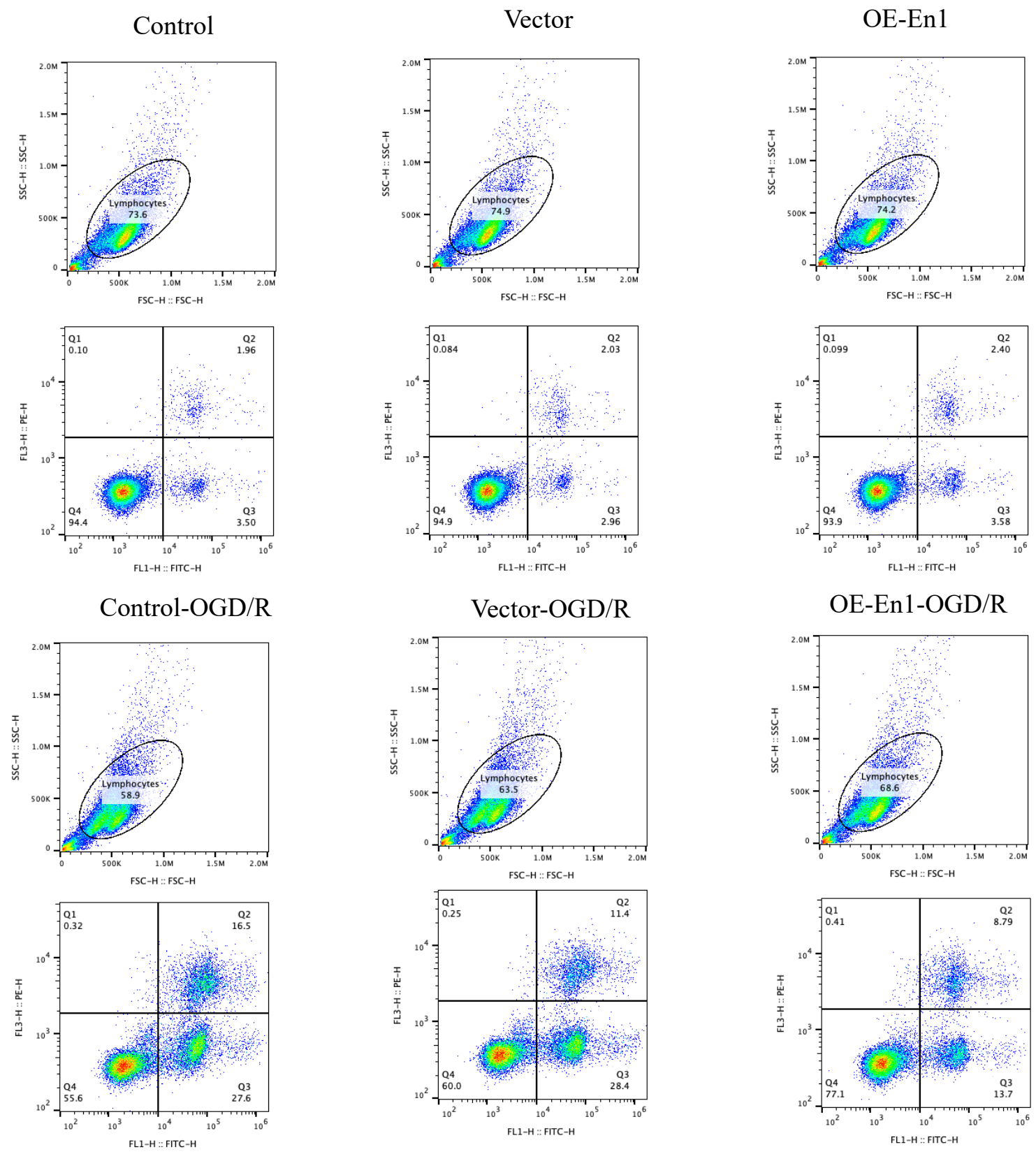

Supplementary Figure 9: FACS Gating Strategy images in Figure 6.

Figure 6H

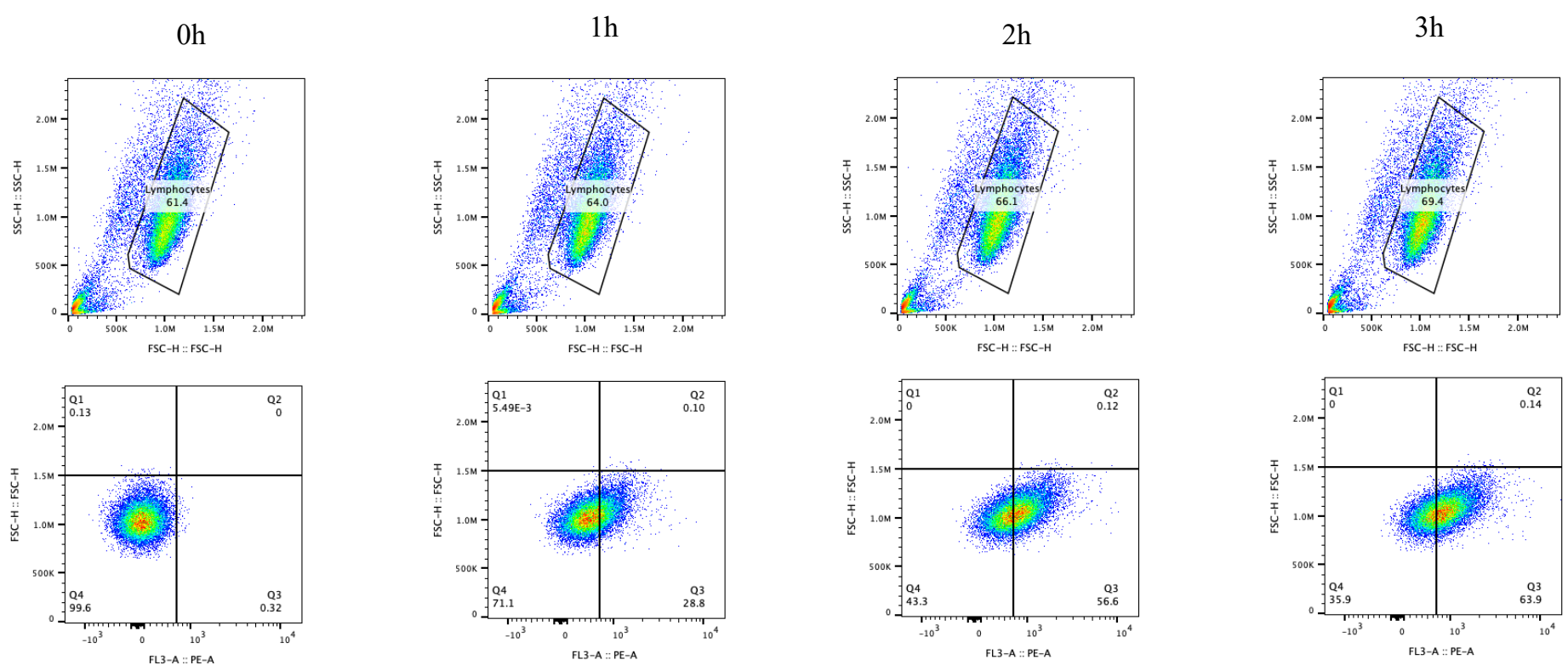

Supplementary Figure 10: FACS Gating Strategy images in Figure 7.

Figure 7E

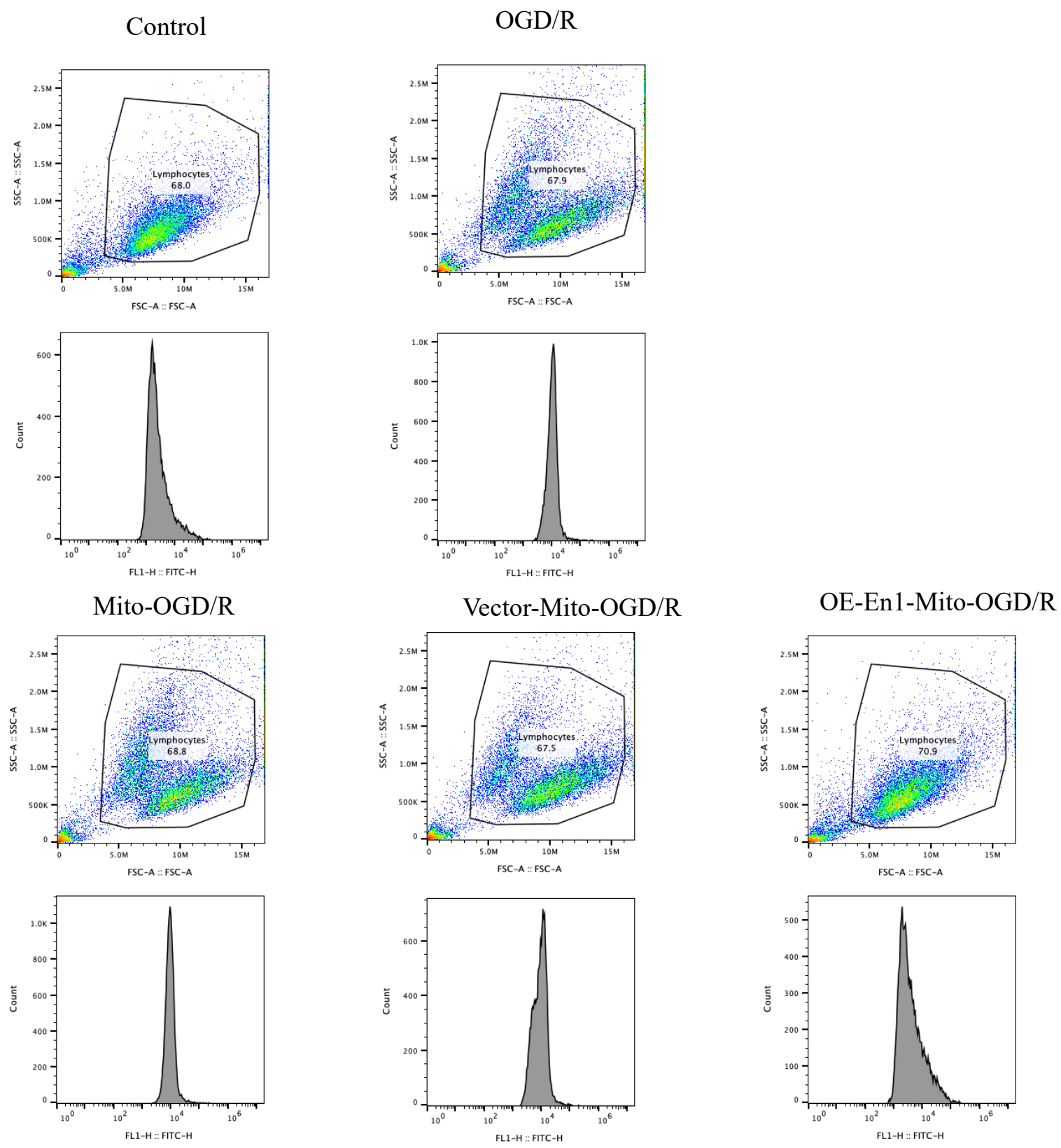

Figure 7G

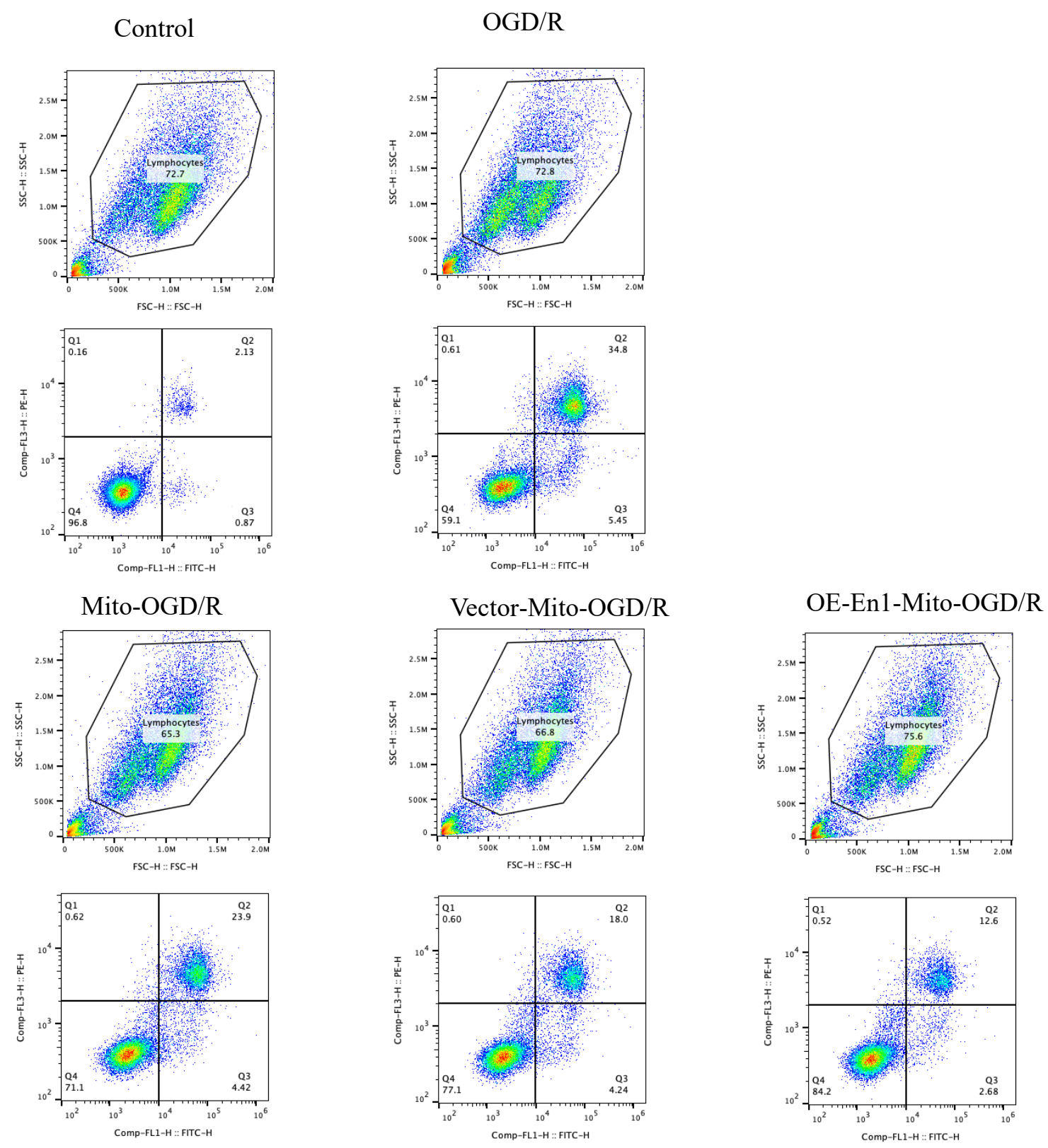

Supplementary Data

Figure 1B

Optical Density	Control	SCI/RI
	0 119	0 109
	0 112	0 111
	0 115	0 107
	0 120	0 100
	0 110	0 105
	0 117	0 106
	0 121	0 112
	0 113	0 104
	0 116	0 109
	0 122	0 105

Figure 1D

Fluorescence intensity Ratio (En-1 (Red) / MAP2 (Green)	Control	SCI/RI
	1 723	1 072
	1 823	1 079
	1 663	1 123
	1 564	0 998
	1 992	1 456
	1 443	1 220
	1 843	1 125
	1 932	1 289
	1 552	1 167
	1 602	1 188

Figure 1F

Relative Protein Expression (En-1/β-actin)	Control	SCI/RI
	0 508	0 164
	0 602	0 123
	0 467	0 225
	0 522	0 180
	0 492	0 199

Figure 1G

Relative mRNA Expression (En-1)	Control	SCI/RI
	1 000	0 670
	1 000	0 550
	1 000	0 500
	1 000	0 620
	1 000	0 480

Figure 1I

Relative Protein Expression (En-1/β-actin)	Control	pre-30min+OGD/R	pre-60min+OGD/R	pre-90min+OGD/R	pre-120min+OGD/R	OGD/R
	0 52	0 22	0 28	0 97	0 59	0 21
	0 55	0 27	0 38	0 69	0 48	0 19
	0 61	0 30	0 36	0 85	0 84	0 25

Figure 1J

Relative mRNA Expression (En-1)	Control	pre-30min+OGD/R	pre-60min+OGD/R	pre-90min+OGD/R	pre-120min+OGD/R	OGD/R
	1 00	1 27	1 13	1 60	1 54	0 46
	1 00	0 92	1 24	1 54	1 25	0 40
	1 00	0 79	0 91	1 78	1 51	0 56

Figure 1K

Cell viability % (OD 450nm)	Control	pre-30min+OGD/R	pre-60min+OGD/R	pre-90min+OGD/R	pre-120min+OGD/R	OGD/R
	100 00	75 60	77 40	90 57	80 33	54 32
	100 00	78 35	83 23	88 72	90 12	49 31
	100 00	66 30	80 93	82 37	89 73	57 23

Figure 2C

Relative Protein Expression (En-1/β-actin)	Control	Vector	OE-En1
	0 10	0 13	0 41
	0 10	0 09	0 45
	0 20	0 10	0 50

Figure 2D

Relative Protein Expression (En-1/β-actin)	Control	Vector	OE-En1
	1 00	1 12	81 19
	1 00	0 97	100 33
	1 00	0 89	66 30

Figure 2E

Cell viability % (OD 450nm)	Control	Vector	OE-En1	OGD/R		
				Control	Vector	OE-En1
	100 00	99 36	98 92	62 79	60 23	79 54
	100 00	99 42	98 12	55 32	54 39	75 30
	100 00	98 24	99 66	52 37	63 28	84 32

Figure 2H

Fluorescence intensity Ratio (Mito-membrane potential Red/Green)	Control	Vector	OE-En1	OGD/R		
				Control	Vector	OE-En1
	11 16	15 32	23 20	0 82	1 26	5 98
	16 62	8 24	27 65	1 23	0 74	12 65
	9 13	13 42	21 70	0 99	1 12	9 23

Figure 2I

High mitochonfrisl potential ratio (%)	Control	Vector	OE-En1	OGD/R		
				Control	Vector	OE-En1
	94 60	92 50	94 10	58 10	61 40	75 80
	92 80	95 40	91 20	55 80	57 10	77 90
	90 30	90 30	95 60	64 20	64 10	70 20

Figure 2L

Mean Fluorescene Intensity ROS Expression	Control	Vector	OE-En1	OGD/R		
				Control	Vector	OE-En1
	6321	6614	4551	18750	17504	9134
	6778	5423	5545	20320	19287	7778
	5790	5724	2784	17560	22342	11234

Figure 3B

Apoptosis rate(%)	Control	Vector	OE-En1	OGD/R		
				Control	Vector	OE-En1
	5 46	4 99	5 98	44 10	39 80	22 49
	8 34	7 82	9 33	49 34	49 50	30 70
	6 73	9 33	4 79	40 25	44 30	35 30

Figure 3D

Relative Protein Expression (Cleaved Caspase 3/Caspase 3)	Control	Vector	OE-En1	OGD/R		
				Control	Vector	OE-En1
	0 45	0 35	0 48	0 57	0 59	0 39
	0 23	0 21	0 32	0 65	0 78	0 43
	0 34	0 15	0 24	0 52	0 62	0 35

Relative Protein Expression (Bax/β-actin)	Control	Vector	OE-En1	OGD/R		
				Control	Vector	OE-En1
	0 16	0 18	0 19	1 18	1 22	0 55
	0 18	0 16	0 21	1 12	1 07	0 73
	0 19	0 17	0 16	1 26	1 14	0 48

Relative Protein Expression (Bcl-2/β-actin)	Control	Vector	OE-En1	OGD/R		
				Control	Vector	OE-En1
	0 91	1 02	0 81	0 11	0 11	0 30
	0 88	0 96	0 94	0 11	0 11	0 40
	0 97	0 96	0 87	0 12	0 11	0 34

Figure 3E

ATP relative content (%/Control)	Control	Vector	OE-En1	OGD/R		
				Control	Vector	OE-En1
	1 00	0 98	1 00	0 56	0 69	0 82
	1 00	0 90	1 02	0 67	0 53	0 80
	1 00	0 92	0 92	0 66	0 61	0 74

Figure 3F

SOD activity (U/mgprot)	Control	Vector	OE-En1	OGD/R		
				Control	Vector	OE-En1
	76	67	70	38	42	63
	65	64	69	49	46	49
	70	74	67	47	39	58

Figure 3G

MDA content (nmol/mgprot)	Control	Vector	OE-En1	OGD/R		
				Control	Vector	OE-En1
	0 55	0 59	0 45	0 79	0 85	0 55
	0 46	0 49	0 52	0 83	0 72	0 68
	0 57	0 51	0 53	0 76	0 79	0 67

Figure 5F

Relative luciferase level	pGL3-Basic+pRL-TK Vector	pGL3-Basic+EN1+pRL-TK Vector	pGL3-Basic-Promoter+pRL-TK Vector	pGL3-Basic-Promoter+EN1+pRL-TK Vector
	1 00	1 21	45 54	60 78
	1 00	1 13	37 75	59 38
	1 00	0 92	36 92	56 59

Figure 5H

Relative Protein Expression (PDGFC/β-actin)	Control	OE-En1	si-PDGFC	OE-En1+si-PDGFC
	0 57	0 84	0 16	0 17
	0 51	0 88	0 20	0 21
	0 54	0 86	0 15	0 19

Relative mRNA Expression (PDGFC)	Control	OE-En1	si-PDGFC	OE-En1+si-PDGFC
	1 00	1 60	0 20	0 35
	1 00	1 52	0 40	0 23
	1 00	1 30	0 12	0 51

Figure 5I

Relative Protein Expression (OPA1/β-actin)	Control	OE-En1	si-PDGFC	OE-En1+si-PDGFC
	0 55	1 00	0 18	0 16
	0 59	0 99	0 20	0 18
	0 53	1 00	0 14	0 20

Relative Protein Expression (MFN1/β-actin)	Control	OE-En1	si-PDGFC	OE-En1+si-PDGFC
	0 36	0 31	0 26	0 23
	0 39	0 34	0 31	0 25
	0 42	0 30	0 25	0 26

Relative Protein Expression (MFN2/β-actin)	Control	OE-En1	si-PDGFC	OE-En1+si-PDGFC
	0 66	0 62	0 48	0 63
	0 52	0 49	0 38	0 50
	0 78	0 74	0 59	0 75

Relative Protein Expression (Drp1-Ser616/Drp1)	Control	OE-En1	si-PDGFC	OE-En1+si-PDGFC
	0 44	0 36	0 34	0 35
	0 35	0 29	0 27	0 28
	0 52	0 45	0 42	0 43

Relative Protein Expression (FIS1/β-actin)	Control	OE-En1	si-PDGFC	OE-En1+si-PDGFC
	0 42	0 38	0 34	0 37
	0 43	0 41	0 38	0 39
	0 40	0 38	0 35	0 33

Figure 5K

Relative Protein Expression (OPA1/VDAC1)	Control	OE-En1	si-PDGFC	OE-En1+si-PDGFC
	0 72	1 38	0 34	0 34
	0 58	1 11	0 27	0 28
	0 87	1 65	0 42	0 41

Relative Protein Expression (MFN1/VDAC1)	Control	OE-En1	si-PDGFC	OE-En1+si-PDGFC
	0 95	0 93	0 96	0 78
	0 78	0 76	0 79	0 64
	1 12	1 10	1 13	0 92

Relative Protein Expression (MFN2/VDAC1)	Control	OE-En1	si-PDGFC	OE-En1+si-PDGFC
	0 53	0 53	0 49	0 38
	0 42	0 43	0 39	0 30
	0 64	0 63	0 60	0 46

Relative Protein Expression (Drp1-Ser616/Drp1)	Control	OE-En1	si-PDGFC	OE-En1+si-PDGFC
	0 84	0 80	0 82	0 96
	0 69	0 66	0 67	0 79
	0 99	0 94	0 97	1 13

Relative Protein Expression (FIS1/VDAC1)	Control	OE-En1	si-PDGFC	OE-En1+si-PDGFC
	0 76	0 81	0 85	0 71
	0 62	0 67	0 70	0 58
	0 90	0 95	0 92	0 84

Relative Protein Expression (β-actin/VDAC1)	Control	OE-En1	si-PDGFC	OE-En1+si-PDGFC
	0 17	0 24	0 18	0 16
	0 14	0 19	0 16	0 13
	0 20	0 12	0 11	0 19

Figure 6C

Fluorescence intensity Ratio (MitoTracker Red/Green)	Control	Vector	OE-En1
	1 26	1 74	1 52
Mito	1 68	1 33	1 82
	1 72	1 45	1 65
	0 32	0 56	0 12
Mito+CCCP	0 14	0 44	0 32
	0 18	0 26	0 30

Figure 6C

Compex (U/mg prot)	Complex I	Complex IV
	1250	1452
Control	1123	1345
	1067	1126
	983	1421
Vector	1289	1089
	1123	1167
	984	1411
OE-En1	1136	1294
	1213	1134

Figure 6D

ATP relative content (%/Control)	Control	Vector	OE-En1
	1 00	0 92	1 12
	1 00	0 97	0 96
	1 00	0 89	0 94

Figure 6G

Fluorescence intensity Ratio (MitoTracker Red/Green)	0 h	1 h	2 h	3 h
	0 32	47 80	56 60	87 50
	0 78	33 50	70 22	68 90
	1 33	40 30	65 60	72 30

Figure 6I

Mitotracker Red positive cells (%)	0 h	1 h	2 h	3 h
	0 32	28 80	56 60	63 90
	0 78	33 50	47 30	68 90
	1 33	22 40	51 20	59 30

Figure 7A

ATP relative content (%/Control)	Control	OGD/R	Mito-OGD/R	Vector-Mito-OGD/R	OE-En1-Mito-OGD/R
	1 00	0 52	0 77	0 72	0 82
	1 00	0 45	0 68	0 69	0 79
	1 00	0 53	0 73	0 74	0 83

Figure 7B

SOD activity (U/mgprot)	Control	OGD/R	Mito-OGD/R	Vector-Mito-OGD/R	OE-En1-Mito-OGD/R
	89	35	51	44	58
	79	39	46	47	61
	77	37	49	49	70

Figure 7C

MDA content (nmol/mgprot)	Control	OGD/R	Mito-OGD/R	Vector-Mito-OGD/R	OE-En1-Mito-OGD/R
	0 34	0 89	0 68	0 71	0 48
	0 44	0 81	0 70	0 75	0 50
	0 39	0 80	0 72	0 66	0 59

Figure 7F

Mean Fluorescene Intensity ROS Expression	Control	OGD/R	Mito-OGD/R	Vector-Mito-OGD/R	OE-En1-Mito-OGD/R
	4686	12609	11535	10800	7592
	3940	13465	10795	11110	8235
	4232	12032	10023	9993	7232

Figure 7H

Apoptosis rate(%)	Control	OGD/R	Mito-OGD/R	Vector-Mito-OGD/R	OE-En1-Mito-OGD/R
	3 01	40 24	28 22	22 24	15 28
	4 12	38 20	25 44	28 93	22 32
	4 60	35 40	32 56	30 23	18 34

Figure 7J

Relative Protein Expression (Cleaved Caspase 3/Caspase 3)	Control	OGD/R	Mito-OGD/R	Vector-Mito-OGD/R	OE-En1-Mito-OGD/R
	0 12	0 78	0 54	0 52	0 21
	0 23	0 82	0 52	0 59	0 15
	0 33	0 72	0 61	0 65	0 34

Relative Protein Expression (Bax/β-actin)	Control	OGD/R	Mito-OGD/R	Vector-Mito-OGD/R	OE-En1-Mito-OGD/R
	0 16	0 86	0 45	0 42	0 34
	0 21	0 97	0 61	0 50	0 29
	0 18	0 76	0 43	0 46	0 31

Relative Protein Expression (Bcl-2/β-actin)	Control	OGD/R	Mito-OGD/R	Vector-Mito-OGD/R	OE-En1-Mito-OGD/R
	0 99	0 20	0 45	0 26	0 51
	0 99	0 14	0 35	0 28	0 64
	1 124	0 164	0 453	0 421	0 688

Figure 8D

Number of Motor Neurons	Control	SCI/RI	Mito-SCI/RI	Vector-Mito-SCI/RI	OE-En1-Mito-SCI/RI
	40	4	21	23	47
	67	15	25	30	45
	54	8	30	26	50
	46	10	31	27	41
	64	17	27	22	39

Figure 8E

Red Positive Cells (%)	Control	SCI/RI	Mito-SCI/RI	Vector-Mito-SCI/RI	OE-En1-Mito-SCI/RI
	0 00	15 24	11 86	10 08	8 19
	0 00	16 32	10 21	12 31	5 21
	0 00	12 13	9 73	7 13	6 77
	0 00	10 23	8 21	10 54	4 43
	0 00	13 22	8 88	9 14	6 03

Figure 8F

Motor Deficit Index 24h after SCI/RI	Control	SCI/RI	Mito-SCI/RI	Vector-Mito-SCI/RI	OE-En1-Mito-SCI/RI
	0	6	4	4	3
	0	6	4	4	3
	0	6	5	5	4
	0	5	5	4	2
	0	6	4	4	3

Figure 8G

Motor Deficit Index 48h after SCI/RI	Control	SCI/RI	Mito-SCI/RI	Vector-Mito-SCI/RI	OE-En1-Mito-SCI/RI
	0	5	4	4	3
	0	5	4	4	3
	0	6	4	4	2
	0	6	5	3	2
	0	6	3	4	2

Figure 8H

Motor Deficit Index 7d after SCI/RI	Control	SCI/RI	Mito-SCI/RI	Vector-Mito-SCI/RI	OE-En1-Mito-SCI/RI
	0	5	4	4	3
	0	4	4	4	2
	0	5	4	4	2
	0	5	4	3	2
	0	6	3	3	2

Figure 9B

Relative Protein Expression (OPA1/β-actin)	Control	OGD/R	Mito-OGD/R	Vector-Mito-OGD/R	OE-En1-Mito-OGD/R
	0 76	0 40	0 82	0 86	1 15
	0 80	0 43	0 88	0 93	1 12
	0 75	0 38	0 83	0 86	1 02

Relative Protein Expression (MFN1/β-actin)	Control	OE-En1	si-PDGFC	OE-En1+si-PDGFC	OE-En1-Mito-OGD/R
	0 45	0 18	0 51	0 56	0 75
	0 51	0 20	0 56	0 59	0 84
	0 43	0 16	0 50	0 54	0 73

Relative Protein Expression (MFN2/β-actin)	Control	OE-En1	si-PDGFC	OE-En1+si-PDGFC	OE-En1-Mito-OGD/R
	0 65	0 42	0 62	0 68	0 86
	0 54	0 36	0 52	0 57	0 72
	0 74	0 48	0 71	0 78	0 98

Relative Protein Expression (Drp1-Ser616/Drp1)	Control	OE-En1	si-PDGFC	OE-En1+si-PDGFC	OE-En1-Mito-OGD/R
	0 28	0 80	0 70	0 60	0 49
	0 19	0 76	0 58	0 72	0 40
	0 35	0 89	0 66	0 63	0 59

Relative Protein Expression (FIS1/β-actin)	Control	OE-En1	si-PDGFC	OE-En1+si-PDGFC	OE-En1-Mito-OGD/R
	0 48	0 97	0 58	0 56	0 45
	0 51	1 02	0 62	0 61	0 49
	0 47	0 93	0 57	0 56	0 39

Figure 9D

Relative Protein Expression (OPA1/β-actin)	Mito	OE-En1-Mito	OE-En1/si-PDGFC-Mito	OGD/R
	0 76	1 00	0 18	
	0 74	0 96	0 20	
	0 64	0 89	0 17	

Relative Protein Expression (MFN1/β-actin)	Mito	OE-En1-Mito	OE-En1/si-PDGFC-Mito	OGD/R
	0 56	0 85	0 44	
	0 61	0 89	0 46	
	0 55	0 79	0 41	

Relative Protein Expression (MFN2/β-actin)	Mito	OE-En1-Mito	OE-En1/si-PDGFC-Mito	OGD/R

	0 55	0 78	0 37
	0 42	0 64	0 25
	0 48	0 71	0 49

Relative Protein Expression (Drp1-Ser616/Drp1)	Mito	OE-En1-Mito	OE-En1/si-PDGFC-Mito	OGD/R
	0 73	0 55	0 76	
	0 59	0 42	0 63	
	0 84	0 48	0 87	

Relative Protein Expression (FIS1/β-actin)	Mito	OE-En1-Mito	OE-En1/si-PDGFC-Mito	OGD/R
	0 57	0 38	0 77	
	0 45	0 25	0 62	
	0 68	0 50	0 89	

Figure 9F

Fluorescence intensity Ratio (OPA1 (Green) / DAPI (Blue)	Mito	OE-En1-Mito	OE-En1/si-PDGFC-Mito	OGD/R
	0 40	1 12	0 23	
	0 34	0 98	0 12	
	0 52	1 34	0 33	
